# In Silico Screening and Testing of FDA-Approved Small Molecules to Block SARS-CoV-2 Entry to the Host Cell by Inhibiting Spike Protein Cleavage

**DOI:** 10.3390/v14061129

**Published:** 2022-05-24

**Authors:** E. Sila Ozdemir, Hillary H. Le, Adem Yildirim, Srivathsan V. Ranganathan

**Affiliations:** 1 Cancer Early Detection Advanced Research Center, Knight Cancer Institute, Oregon Health & Science University, Portland, OR 97201, USA; lehi@ohsu.edu (H.H.L.); yildirim@ohsu.edu (A.Y.); 2 Division of Oncological Sciences, Knight Cancer Institute, Oregon Health & Science University, Portland, OR 97201, USA

**Keywords:** drug repurposing, SARS-CoV-2, molecular modeling, in silico screening, proteases

## Abstract

The COVID-19 pandemic began in 2019, but it is still active. The development of an effective vaccine reduced the number of deaths; however, a treatment is still needed. Here, we aimed to inhibit viral entry to the host cell by inhibiting spike (S) protein cleavage by several proteases. We developed a computational pipeline to repurpose FDA-approved drugs to inhibit protease activity and thus prevent S protein cleavage. We tested some of our drug candidates and demonstrated a decrease in protease activity. We believe our pipeline will be beneficial in identifying a drug regimen for COVID-19 patients.

## 1. Introduction

The outbreak of the novel severe acute respiratory syndrome coronavirus 2 (SARS-CoV-2) in China was followed by a global level emergency. COVID-19, the disease caused by SARS-CoV-2, is a global pandemic and has resulted in over six million deaths, and the death toll continues to climb, which is devastating, as the virus is expected to be in circulation for a long time [1]. The novel coronavirus has been sequenced, and it has been revealed that it shares substantial similarity with the SARS-CoV-1 that caused a similar epidemic in 2003 [2]. They are both zoonotic coronaviruses belonging to the betacoronavirus family [3].

The world is in search of a robust drug with universal applicability and high efficacy, which can be scaled rapidly to high levels of production in order to fight this virus family. Considering the fact that trials and approval processes of novel, designed small molecules take approximately 10 to 15 years in the US [4], it would be a wise choice to repurpose the already well-characterized small molecules to respond to this emergency.

To date, there has been a significant amount of work carried out to test previously approved antiviral drugs, resulting in a few successes, such as Remdesivir by Gilead and Molnupiravir by Merck, with modest levels of improvement in treatment outcomes. However, there is still a need for a more comprehensive treatment of SARS-CoV-2 to further decrease the fatality and minimize the spread of the virus.

**Figure 1:**
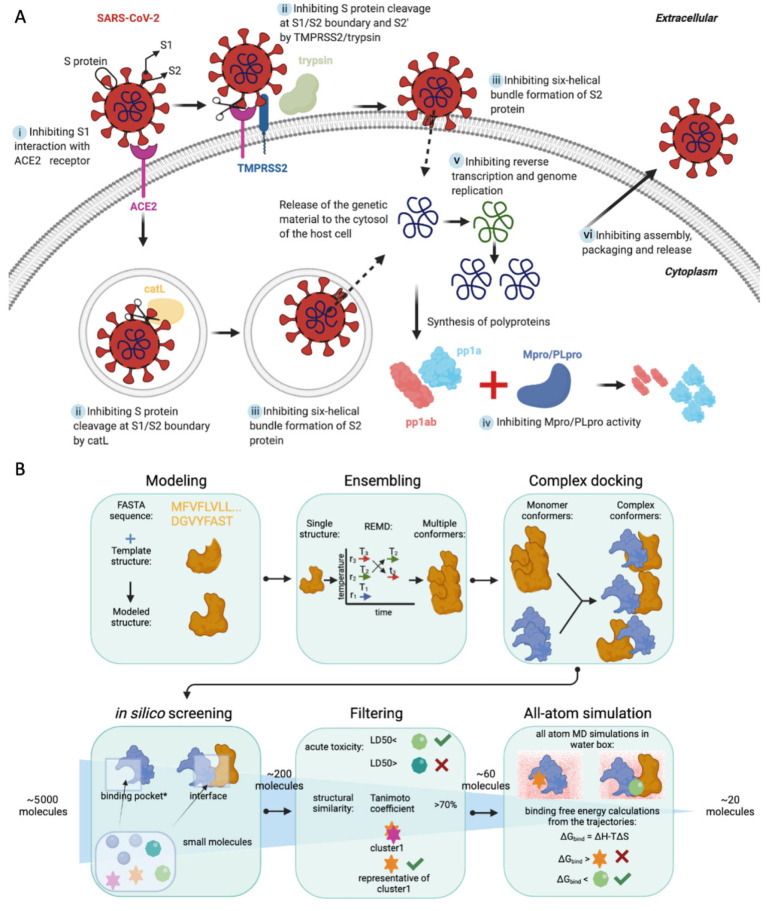
The targeting strategy and pipeline. (**A**) A summary of viral protein targeting strategies. Among six main strategies denoted here as i, ii, iii, iv, v and vi, we focused on ii. (**B**) The computational pipeline flow used for screening drugs starting from the target FASTA sequence to the bench. r in the ensembling panel stands for replica, while T in the same panel stands for temperature. Binding pockets of the monomers correspond with the interface sites of each binding partner in the complex. The number of small molecules at the beginning of the in silico screening and in-between each step is written before and after the steps. In silico screening starts with 5000 molecules; this number decreases to 200 after the screening. Then, filtering narrows this number down to 60 molecules. MD simulations and binding free energy calculations result in 20 molecules to be evaluated on the bench. This figure is created with BioRender.

As an early therapeutic strategy, there are already a number of studies to identify small molecules to inhibit SARS-CoV-2 activity in the host cells. To our knowledge, there are six main strategies/routes to target the viral proteins [5] (Figure 1A): (i) Inhibiting the interaction between the viral spike (S) protein and angiotensin-converting enzyme 2 (ACE2) receptor of the host [6,7]. The initial attachment of the virus to the host cell is initiated by this interaction [8]. (ii) Inhibiting S protein cleavage by proteases. Following receptor binding, the virus gains access to the host cell cytosol by acid-dependent proteolytic cleavage of S protein by TMPRSS2, trypsin, cathepsin L (catL) or other proteases at the S1/S2 boundary and S2’ [8]. (iii) Blocking the entry of the virus into the cytosol by inhibiting the fusion of virus to the endosomal membrane. Following proteolytic cleavage, the heptad repeat 1 (HR1) and heptad repeat 2 (HR2) regions of the S2 domain interact to form a six helical bundle, which plays an important role in the fusion to the membrane. HR1 and HR2 regions can be targeted to prevent viral entry into the host cell [9]. (iv) Inhibiting the proteases essential for proteolytic processing of viral polyproteins [10,11]. After entering the host cell, the virus continues its lifecycle by translating its genes, which express two co-terminal polyproteins, pp1a and pp1ab. Proteolytic processing of viral polyproteins by the main protease (Mpro) and papain-like protease (PLpro) results in nonstructural proteins, which basically inhibit most of the function of the host cells. (v) Inhibiting reverse transcription of the viral genomic material and its multiplication [12]. Finally, (vi) Inhibiting assembly, packaging and release of newly formed viruses [12].

Here, we focused on inhibiting viral entry (route ii, Figure 1A) by performing a structure-based screening of FDA-approved drug compounds against the proteases trypsin, catL and TMPRSS2, as well as the viral S protein. S protein consists of two main domains: S1 domain responsible for receptor binding and S2 domain mediating membrane fusion. In the case of SARS-CoV, the S protein is proteolytically cleaved at the S1/S2 boundary [13]. However, recent studies have revealed that cleavage at a secondary site (S2’) is also critical for viral activation by serine proteases, such as TMPRRS2 and trypsin, and entry into the host cell [13,14]. Therefore, we aim to identify small molecules to be repurposed to disrupt S protein cleavage at both S1/S2 boundary and S2’. Even though the SARS-CoV endosomal entry requires catL protease [15], it has been shown that TMPRRS2-mediated activation, governed by the S2’ site, is required for viral pathogenesis [16,17].

Our approach was to target either the S protein and/or proteases to inhibit both endosomal (catL) and TMPRRS2-mediated entry of the virus. Recent efforts have already produced all-atom structures of the novel SARS-CoV-2 S protein, showing a well-established structural region at S2’, which can be deployed to perform molecular drug screening. Furthermore, this secondary site is conserved between the SARS variants (unlike the primary site) and therefore allows for broader applicability against other strains of the virus.

In this study, we obtained structural ensembles of protease–S protein complexes that capture the inherent configurational diversity using replica exchange molecular dynamics (REMD) simulations and use those structures for in silico small molecule screening. This approach allows us to explore thermally accessible configurations other than just the lowest energy structure, which are likely to play important roles in molecular recognition [18]. In addition to targeting the S protein from the original strain, we also included a recently observed S protein mutation (D936Y) in our analyses, which is predicted to have a significant impact on the structure of the protein [19]. This mutation is identified as a result of analysis of the HR1 mutation occurrences in 34,805 SARS-CoV-2 genomic sequences in GISAID (19). The study revealed a dramatic increase in the D936Y mutation, which was particularly widespread in Sweden and Wales/England. We spotted this mutation as close to the trypsin binding site of S2’, and therefore, we obtained ensembles of configurations for trypsin–S protein^D936Y^ complex.

We used the structural ensemble of the proteins to screen approximately 5000 small molecule structures. These structures were curated from various drug libraries, and they were all FDA-approved small molecular drugs. However, the recurring structures occurring in more than one database were not removed, and different confirmations of the same small molecular drugs were included in the screening. From this ensemble docking procedure, we ranked the drug molecules based on predicted binding affinities to structures of unbound proteases and S proteins, as well as protease–S protein complexes, and we identified the top ~200 molecules with <−7.4 kcal/mol binding free energies. Furthermore, we filtered down the list to 55 molecules by clustering based on chemical, structural and functional similarity, and eliminating molecules with high toxicities. The top 55 molecules were subject to all-atom molecular dynamics (MD) simulations to assess the stability of the interaction in explicit water, and for stable complexes, binding free energies were estimated using the molecular mechanics Poisson–Boltzmann surface area (MM-PBSA) method to produce a list of hits. Finally, we tested our hit candidates experimentally, at least in one case (against catL), for their potential to inhibit protease activity. Indeed, the experiments revealed catL inhibition for two of the five molecules that were predicted in silico, highlighting the potential of this pipeline (Figure 1B) for high-throughput drug screening.

## 2. Results

**Figure 2:**
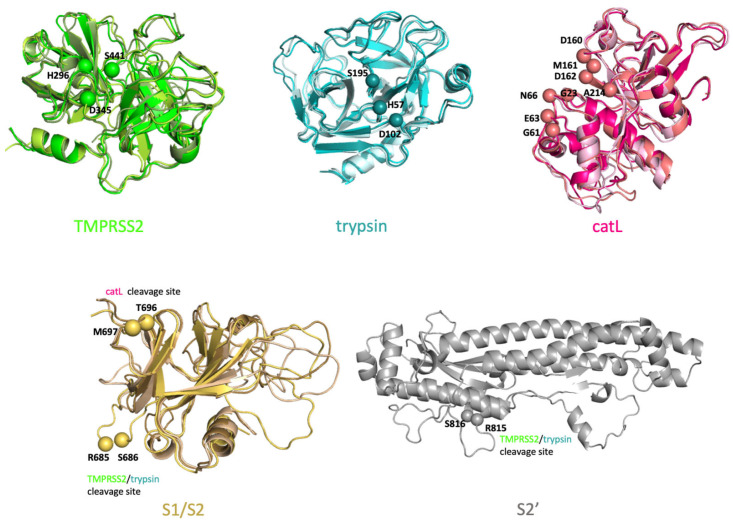
Ensembles of configurations of unbound proteases and spike (S) protein cleavage sites. Three configurations for TMPRSS2, trypsin, catL and S1/S2, as well as one configuration for S2’, were used for in silico screening. The catalytic residues on TMPRSS2, trypsin and catL, as well as the cleavage sites of S protein structures, were labeled, and Cα atoms of the residues were represented with spheres.

**Figure 3:**
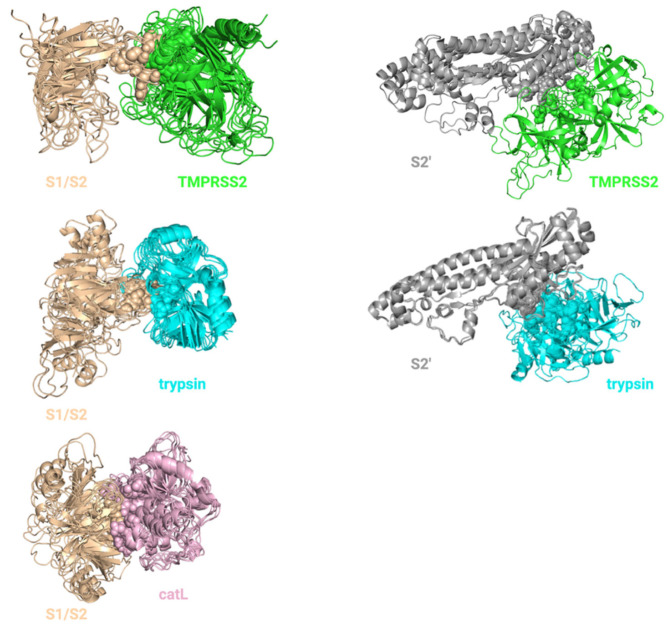
Ensembles of configurations of protease–S protein complexes. Four configurations for TMPRSS2-S1/S2 complex, two configurations for TMPRSS2-S2’ complex, six configurations for trypsin-S1/S2 complex, three configurations for trypsin-S2’ complex, six configurations for catL-S1/S2 complex were used for in silico screening. Catalytic sites of the proteases and cleavage sites of the S protein are shown with spheres.

To perform docking, we generated an ensemble of receptor configurations using REMD simulations. To that end, we performed root-mean-square deviation (RMSD) clustering of the structures obtained from the simulations and chose the central structures from the top three clusters, which accounted for more than 70% of all conformational states (Figure 2 and Figure 3). Over 205,000 docking calculations were then performed to dock 5000 small molecules to our ensembles of configurations for our 17 monomer and 24 complex structures. The top 20 hits for each structure were selected. Below, we discussed specific results of docking calculations for monomer and complex structures, separately.

### 2.1. Evaluating the In Silico Docking Pipeline Incorporating an Ensemble of Receptor Configurations

**Figure 4:**
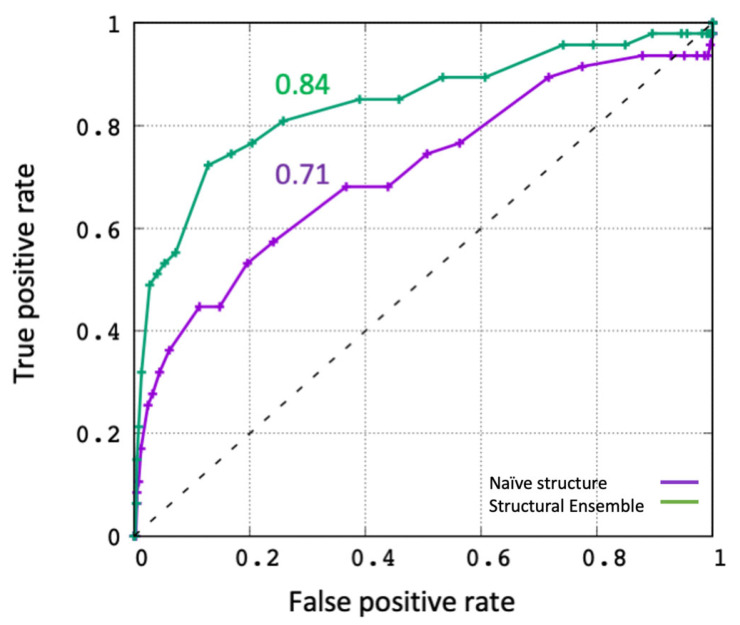
Receiver operating curves (ROC) to evaluate performance of the in silico screening. Docking of active (47) and decoy (1652) molecules was performed against the crystal structure of Trypsin (purple) and against the structural ensemble generated by REMD simulations (green). The area under the curve (AUC) values are also shown.

To evaluate the performance of this method, we took advantage of the Directory of Useful Decoys (DUD) dataset [20], which contains a validated list of active and decoy compounds for a number of proteases, including one of our targets (Trypsin). Using the ensemble generated for Trypsin, we were able to dock the active (47) and decoy molecules (1652) and compare the performance against docking to the crystal structure, which we refer to as the naïve method. The results are summarized in terms of a receiver operating curve (ROC) shown in Figure 4. The naïve method performs decently, as characterized by an area under the curve (AUC) of 0.71, which is in good agreement with previously published performance of Vina (AUC = 0.72) [21]. Remarkably, the ensemble method showed a significant improvement in performance with an AUC of 0.84, aided by molecular simulations in explicit water. Therefore, we proceeded with this ensemble method for screening of the drugs against our protease and spike protein targets. In the future, we plan on validating this method with a wide range of targets from the DUD database to comprehensively evaluate its performance.

### 2.2. Identifying the Top Molecules to Target Unbound Proteases, S Protein and Protease-S Protein Complexes

The S2’ site of the S protein was not subject to REMD simulations, as the target region is a rigid alpha helix bundle. Therefore, the ensemble of monomer receptors comprised a total of seventeen structures, as listed: three each of trypsin, catL, TMPRSS2, S1/S2-a, S1/S2-b, and one of S2’ and S2’_D936Y. S1/S2-a refers to docking against S1/S2 boundary cleavage site targeted by TMPRSS2 and trypsin (R685-S686), while S1/S2-b indicates docking against S1/S2 boundary cleavage site targeted by only catL (T696-M697). S2’, by itself, is the secondary cleavage site on the S protein targeted by TMPRSS2 and trypsin.

In total, 85,000 docking calculations using the 17 conformations of the S protein and proteases were performed, and the top 19–21 hits for each ligand were listed in Table S1. The docking scores varies from −13.6 to −7.4. From the list, 96 distinct molecules (hereinafter referred to as ligands) were identified targeting either/both S protein cleavage sites or/and catalytic sites of proteases. Considering the similarity between the catalytic sites of TMPRSS2 and trypsin, we expected to see some common molecules targeting both of these proteases, as seen in the table.

The total of 24 complex structures are as follows: trypsin_1-S1/S2_1, trypsin_2-S1/S2_1, trypsin_3-S1/S2_1, trypsin_1-S1/S2_2, trypsin_2-S1/S2_2, trypsin_3-S1/S2_2, trypsin_1-S2’_1, trypsin_2-S2’_1, trypsin_3-S2’_1, trypsin_1-S2’_D936Y_1, trypsin_2-S2’_D936Y_1, trypsin_3-S2’_D936Y_1, catL_1-S1/S2’_1, catL_2-S1/S2’_1, catL_3-S1/S2’_1, catL_1-S1/S2’_2, catL_2-S1/S2’_2, catL_3-S1/S2’_2, TMPRSS2_1-S1/S2_1, TMPRSS2_2-S1/S2_1, TMPRSS2_1-S1/S2_2, TMPRSS2_2-S1/S2_2, TMPRSS2_1-S2’, TMPRSS2_2-S2’. Here is an example to clarify the nomenclature: trypsin_1-S1/S2_1 refers to the complex between the first configuration of trypsin and the first configuration of S1/S2 boundary domain of the S protein.

In total, 120,000 docking calculations using these 24 complexes were performed. No arbitrary docking score cutoff was used for the selection, but the top 19–21 hits for each ligand were listed in Table S2. The docking scores varies from −13.7 to −9.2. From the list, 151 distinct ligands were identified targeting the S protein–protease interaction interfaces.

### 2.3. Curating a More Refined List of Ligands according to Their Toxicity, Structural and Functional Similarity

From the 96 and 151 distinct ligands from in silico screening results for the monomer and the complex structures, respectively, we used three different selection criteria to arrive at a more refined list of ligands. The first selection criterion is based on the toxicity of the ligands. As mentioned before, FDA-approved drugs were used in this study to meet the urgent need for a treatment. To further pursue this approach, the ligands with high acute toxicity rate (such as chemotherapeutics), which cannot be delivered at high concentrations, were given the least preference. The second and third selection criteria are based on the structural and functional similarities of the ligands, respectively. Chemical clustering of ligands was performed according to their Tanimoto coefficients with a similarity cutoff of 70%. Furthermore, the mechanism of action was assigned to each ligand. For example, Tyrosine kinase inhibitors, NS5A inhibitors, ergosterol binders were among the most commonly occurring action mechanisms of the ligands. Finally, the least toxic ligands with a unique mechanism of actions were selected from each cluster. Atopical ligands were eliminated. The refined list of 55 ligands with their LD50s and functions is shown in Table 1. Interestingly, drugs previously proposed or/and used for COVID-19 treatment, such as Remdesivir, Gleevec, Saquinavir, Digoxin, Camostat, Drospirenone, Dexamethasone M and Dihydroergotamine, are among these 55 ligands. However, their potential for blocking viral entry needs to be further explored.

### 2.4. Identifying the Best Binding Ligands to Inhibit Proteolytic Cleavage of S Protein

**Figure 5:**
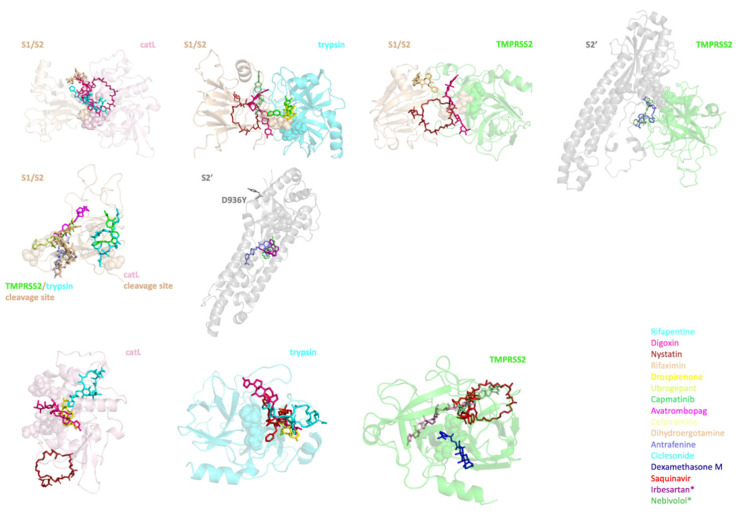
The ligands in complex with their targets. The initial configurations of the ligands bound to their targets are shown in stick representation. Their color-coded list can be seen on the right. Catalytic sites of the proteases and cleavage sites of the S protein are shown with spheres. Two different cleavage sites on the S1/S2 region of the S protein are labeled on the figure. * Irbesartan and Nebivolol are specific to D936Y variant of the S protein.

Considering both TMPRSS2 and trypsin are serine proteases, some of the 55 ligands were targeting more than one complex or monomer. There were 173 initial configurations consisting of ligand–monomer or ligand–protease–S protein complexes. Atomistic MD simulations were performed on these complexes for 100 ns. Using a MMPBSA energy cutoff (ΔG < 0) (Table S3), we identified a small number of ligands for each protease and the S protein to specifically inhibit proteolytic cleavage of the S protein (Table 2 and Figure 5). The initial docked structures of the ligands listed in Table 2 can be seen in Figure 5, which shows the positioning of the ligands in the interfaces of the complex or cleavage/catalytic sites of the S protein/proteases. It is important to note that, although the directions of some ligands seen in Figure 5 might be different than others, they are still around the active sites represented by spheres. Additionally, during the MD simulations, the ligand positions are continuously rearranging, so that they can adapt the most energetically favorable confirmation.

**Figure 6:**
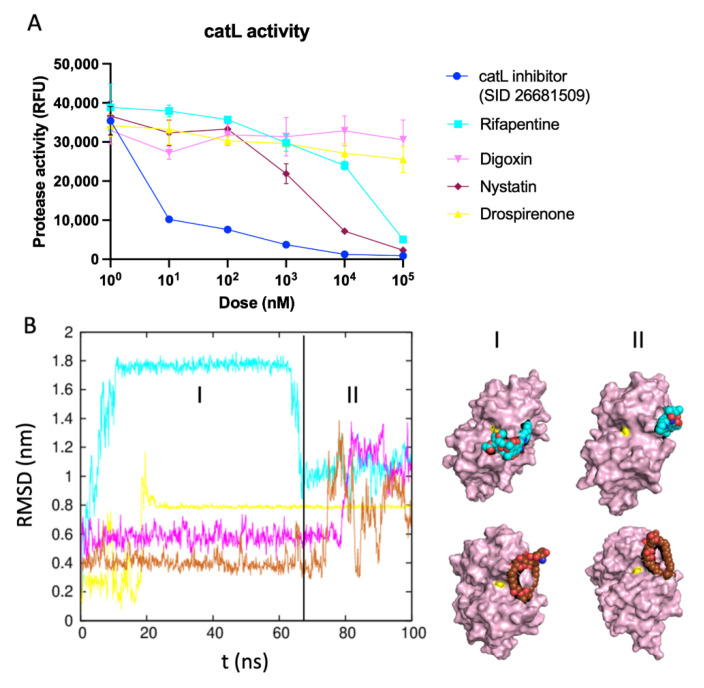
Changes in protease activity of catL upon introducing our proposed small molecules and mechanistic explanation of their effects. (**A**) catL protease activity upon addition of SID 26681509, Rifapentine, Digoxin, Nystatin and Drospirenone. RFU stands for relative fluorescence units. (**B**) RMSD trends of tested drugs (Rifapentine, Digoxin, Nystatin and Drospirenone) in complexes with catL. On the right, two snapshots of our hit drug candidates, Rifapentine (cyan) and Nystatin (brown), in complex with catL (pink) from the start (I) and the end (II) of the simulations are shown. This suggests that the interaction of Rifapentine and Nystatin with the catL active site (shown with yellow sphere) stabilizes the whole complex.

The in silico screening approach allowed us to narrow down the possible viral inhibitors from ~5000 to ~20, which can be tested experimentally for their efficacy to block viral entry into host cells. To demonstrate the proof of concept, we proceeded to validate some of our hits experimentally, at least in one case. We designed an in vitro protease activity assay for catL and observed the possible inhibitory effects of the four drug molecules that are predicted to interact with the active site of the proteases. We introduced the four putative drugs and a known inhibitor (as positive control) over a range of drug concentrations. As expected, the known inhibitors had a significant decrease in fluorescence activity. Excitingly, we found that Nystatin and Rifapentine had measurable effects on the activity of catL. Specifically, Nystatin had an IC50 of ~2 μM and Rifapentine had an IC50 of ~30 μM for inhibiting the protease activity (Figure 6A). In our handful of hits, we found at least two molecules that showed potential in inhibiting protease activity in μM concentrations. These results are also corroborated by the MD simulations, as evidenced by the RMSD plots of the drugs bound to catL (Figure 6B). Interestingly, Nystatin exhibits most stability bound to the protease indicated by the lowest RMSD for the majority of the simulations. While we observed configuration rearrangement toward the end of the simulation, it still remained in close proximity to the active site (the catalytic cysteine is shown in yellow). While Rifapentine shows a large jump in the RMSD from the initial configuration, it is interesting to note that in the higher RMSD state, the molecule resides close to the active site and flips out of the active site while still being bound to the protease. This behavior offers a potential molecular explanation for partial/weaker inhibition of the protease by Rifapentine. The RMSD plots of catL (Figure S2) show minimal perturbation of the protein structure with RMSD remaining <2Å during the course of the simulations. Furthermore, the RMSF plots reveals three distinct flexible regions: 40–50, 90–110 and 170–180, which do not overlap with the active sites. We also investigated the interaction sites by calculating the average number of contacts of catL residues and the drug molecules (Rifapentine, Drospirenone, Digoxin and Nystatin). The residues that showed at least one contact on average are listed in Table S4. Furthermore, we listed the residues with over 70% occupancy, shown in Table S5. Consistent with the inhibition of protease activity (Figure 6A), Nystatin shows the most overlap in interactions with the catL catalytic site.

## 3. Discussion

While the vaccines have provided adequate immunity against the virus, new variants, such as Omicron, with several mutations in the spike protein can be handled comprehensively by establishing a therapeutic strategy that will remain effective against future variants. TMPRSS2, trypsin and catL are considered as potential targets to inhibit SARS-CoV infection dating back to 2003 [22,23]. The inhibition of proteolytic cleavage of the S protein of SARS-CoV-2 by those proteases emerges as crucial, since (1) it prevents the viral genomic material release to the host cell cytoplasm, and (2) it is effective for both extracellular and endosomal viral entry.

In this study, we used homology modeling and REMD to generate an ensemble of configurations for ensemble docking. This approach enabled us to sample a wide variety of conformations of the target proteins (S protein and proteases). The structures chosen for docking following RMSD clustering accounted for more than 70% of the conformational states sampled, which produced the diversity needed for ensemble docking. We screened 5000 small molecules against our ensembles of configurations for our 17 monomer and 24 complex structures, summing up to over 205,000 docking calculations. Following docking, we narrowed down the hits by filtering them based on acute toxicity (LD50) and Tanimoto coefficient of our ligands to identify unique ligands with low toxicity, leading us to a refined list of 55 drugs. This narrowing down allowed us to study the 55 drugs in complex with their targets (173 complexes) in more detail, using all-atom explicit water MD simulations to assess the stability of the interaction and quantify the binding energy in the presence of the solvent explicitly. The RMSD plots and the binding energies from the MD simulations were used to finally rank the drug–target interactions, which allows further experimental investigation of a short list of drugs for their antiviral potential.

As we mentioned, SARS-CoV-2 variants can acquire mutations that increase their virulence, such as enhanced binding to the ACE2 receptor. Therefore, if the treatment regimen involves targeting the ACE2 binding domain of the S protein, its efficacy might decrease when the mutations alter the binding to the drug. In this study, we targeted the human proteases and cleavage sites of the S protein, which tend to be conserved even in the variants. Nevertheless, we identified one variant with a mutation D936Y on the S protein, which is close to the S2’ cleavage site. We modeled the variant with the mutation and identified Irbesartan and Nebivolol as potential drugs specific to the D936Y variant.

The computational pipeline allowed us to screen a large number of drugs and narrowed the list of interest to a few that can be tested at the bench. Two of the four drugs, Nystatin and Rifapentine, showed measurable inhibitory effects on catL activity. Nystatin also emerged as occupying catL catalytic residues (Table S4). Nystatin is an antifungal that is usually used to treat fungal infection in mouth, while Rifapentine is an antibiotic used for treating tuberculosis. It is important to note that these drugs have not been tested for their potential for treatment of COVID-19, and the authors recognize such premature applications of the drug can be harmful and result in shortage of the drugs for patients who need them currently. We are hoping that extensive testing of these drugs for their potential to treat COVID-19 is conducted together with the other recently proposed potential proteolytic inhibitory molecules [24,25], starting from in vitro studies for their potential to block viral entry.

In this study, we generated a computational pipeline for screening drugs from the target FASTA sequence to the bench (Figure 1B). The combination of the computational and experimental techniques enabled us to screen all the FDA-approved drugs and identify some drugs that can effectively target our proteins of interest. Our data recommend a deeper investigation of around a dozen drugs for their potential to inhibit endosomal (catL)-mediated entry of the virus. Many effective vaccines are being applied worldwide, and they significantly reduce the number of new cases in countries with rapid vaccination rates. However, a treatment is still needed, since the vaccine protection is not 100%, and there is inequitable vaccine distribution across the world. Therefore, we believe our proposed regimen will help meet this need.

## 4. Materials and Methods

### 4.1. Computational Methods

#### 4.1.1. Initial Configurations of SARS-CoV-2 S Protein, TMPRSS2, Trypsin and catL

The crystal structure of SARS-CoV-2 spike ectodomain has already been solved (PDB ID: 6vyb [26]); however, it has some unstructured missing region between residues 677 and 690. One of the cleavage sites (R685/S686) are within this missing region. Therefore, the structure of whole-length S protein was modeled using SWISS-MODEL web server [27]; the crystal structure, 6vyb, and FASTA sequence (NCBI Reference Sequence: YP_009724390.1) of SARS-CoV-2 S protein were used as template. S1/S2 boundary and S2’ structures were adapted from our whole-length S protein model. S1/S2 boundary structure covered residues from 293 to 326 and from 591 to 700. S2’ structure consisted of residues from 715 to 1070 (Figure 2, bottom panel).

TMPRSS2 extracellular domain was modeled giving Hepsin crystal structure (PDB ID: 1z8g [28]), a closely related serine protease with more than 50% sequence identity, and FASTA sequence of TMPRSS2 (UniProt ID: O15393) as template using SWISS-MODEL.

The QMean score and Ramachandran plots for the modeled S protein and TMPRSS2 (Figure S1) indicated that our model structures were of high quality to be further used in our analysis.

catL and trypsin crystal structures were already available in PDB (PDB ID: 2xu1 [29] and 1h4w [30], respectively), and they were used in this study.

#### 4.1.2. Replica Exchange MD Simulations

REMD simulations were performed to generate an ensemble of configurations for subsequent protein–protein docking. Simulations were performed using GROningen MAchine for Computer Simulations (GROMACS-2018) on an Exacloud cluster at Oregon Health & Science University (Portland, OR, USA).

The protein structures were centered in a 3D periodic cubic box with initial dimensions ~ 7–8 nm in the three orthogonal directions. The box was then solvated with ~ 12,000 to 14,000 water molecules, depending on the size of the box, and a few Na^+^ or Cl^−^ counterions, depending on the charge of the protein, to keep the system charge neutral. After an initial equilibration of 1 ns, a REMD schedule was set up with 60 windows in the temperature range of 300–425 K. The temperature schedule was generated using the webserver (http://virtualchemistry.org/remd-temperature-generator/ (accessed on 21 May 2020)). The REMD simulations were then performed on the 60 windows for 15 ns with exchanges between neighboring windows tried every 0.4 ps and the protein co-ordinates stored every 50 ps. The first 5 ns were ignored, and the last 10 ns, consisting of 200 frames, were used for clustering analysis.

Clustering was performed using the algorithm described by Daura et al. [31] by using a 5Å RMSD cutoff. Top three configurations from each ensemble covering more than 70% of all configurations were used (except for S2’) for in silico screening of three unbound protease structures and one S1/S2 boundary structure. RMSD values within each ensemble varied from 0.9 to 1.3 Å. For the screening of S2’, one equilibrated configuration was used (Figure 2).

#### 4.1.3. Ensemble Docking for S Protein–Protease Complex Modeling

The configurations generated by REMD for two cleavage domains of the S protein (S1/S2 and S2’), TMPRSS2, trypsin and catL were used to obtain ensembles of configurations for each S protein–protease complex. Top two configurations from S1/S2 boundary and TMPRSS2 ensembles and top three configurations from trypsin and catL ensembles covering ~70% of all configurations were selected for the docking. RMSD values within each ensemble varied from 1.9 to 5.9 Å. One equilibrated S2’ configuration was used. TMPRSS2 and trypsin were docked to both S1/S2 boundary (R685/S686) and S2’ (R815/S816), while catL was only docked to a different cleavage site on the S1/S2 boundary (T696/M697) using HADDOCK web server [32] (Figure 3). Active residues for flexible docking were defined as follows: catalytic triad of TMPRSS2 (H296, D345 and S441) and trypsin (H57, D102 and S195), active sites of catL (G23, G61, E63, N66, D160, M161, D162 and A214) identified in Fujishima et al. [33] and cleavage sites on the S1/S2 boundary (R685/S686 or T696/M697) and S2’ (R815/S816). The cleavage sites on the S1/S2 boundary and S2’ for TMPRSS2, trypsin and catL were obtained by aligning protein sequences of SARS-CoV S protein (UniProt ID: P59594) and SARS-CoV-2 S protein. The sequences of previously defined cleavage sites on SARS-CoV [14] were conserved in SARS-CoV-2, but residue numbers were different (Table 3). In order to obtain S2’^D936Y^ monomer and S2’^D936Y^ -trypsin complexes, PyMol (version 2.3.2) built-in Mutagenesis tool was used.

#### 4.1.4. Small Molecule Libraries and Structure Based in Silico Screening

Clinically approved small molecules from the following drug libraries were used for screening: ZINC library of ~1650 molecules [34], DrugBank of ~ 2500 molecules [35], The Binding Database of ~1340 molecules [36] and ChemBridge of ~50 molecules (top 50 hits from Elshabrawy et al. [15]) obtained from Chembridge Corporation (San Diego, CA, USA). All SDF files from each library were converted to PDBQT using Open Babel software [37].

AutoDock Vina (version 1.1.2, Linux) [38] was used for the docking of small molecules to our configurations. Two approaches were followed for the docking process. First, a total of 17 configurations of unbound proteins (16 WT S protein and protease monomers, and 1 S protein ^D936Y^ monomer) from REMD were used for in silico screening to study the binding of small molecules to the unbound receptors. Moreover, 24 S protein–protease complexes (21 WT S protein–protease complexes and 3 S protein^D936Y^ –proteases complexes) obtained by flexible docking were used for the screening. These two approaches allowed for in silico screening against each PPI partner individually at the interface site, as well as an opportunity to evaluate the possibility of the small molecules destabilizing S protein–proteases complexes. Hydrogens were added to all receptors, and grid box dimensions were defined specific to the size of receptor binding pockets or interfaces (Table S6).

#### 4.1.5. Filtering

The filtering of our ~160 hits was performed using chemical similarity and toxicity. Chemical similarity was quantified using the Tanimoto coefficient, as calculated using the molecular operating environment (MOE). The acute toxicity, as quantified by the mice lethal dose (LD50), was downloaded from FDA drug reports and DrugBank database and listed in Table S7. The mechanism of action data were curated from the PubChem database [39]. The least toxic drugs were selected from clusters that were generated using a 70% chemical similarity, yielding a list of ~55 molecules.

#### 4.1.6. Force Field Generation

All-atom AMBER-type force field parameters were generated for the 55 hit molecules. We created a pipeline to start from a pdb file of the hit molecules to produce gromacs type forcefields for the protein–drug complexes. This pipeline mostly included AMBER tools [40] to calculate partial charges at HF/6-31G (AM1-BCC) [41] and use the General AMBER Force Field (GAFF) [42] for bonded and van der Waals interactions, after ensuring the right protonation state for the molecules.

#### 4.1.7. All-Atom MD Simulations

A total of 173 initial configurations of small molecule–protein complexes (Table S3) were simulated for 100 ns using the GROningen MAchine for Computer Simulations (GROMACS-2018) on an exacloud cluster at Oregon Health & Science University (Portland, OR, USA). MD simulations were carried out in an aqueous environment. The TIP3P water model was used to solvate the system in an aqueous environment with proper number of counterions (Na_+_ or Cl_−_) to ensure charge neutrality. A 3D periodic box was used to center the complex with at least 1.0 nm from the edge, accounting for >2 nm of solvent buffer. The equilibration and production runs were run in NPT ensemble, where the temperature was maintained at 300 K, and the pressure was maintained at 1 bar, using V-rescale thermostat [43] and Parrinello–Rahman barostat, respectively. The MD simulations incorporated a leap-frog algorithm with a 2 fs time step to integrate the equations of motion. The long-ranged electrostatic interactions were calculated using particle mesh Ewald (PME) [44] algorithm with a real space cutoff of 1.2 nm. LJ interactions were also truncated at 1.2 nm. To monitor the systems to reach equilibration, root-mean-square deviations (RMSD) of the complex structures were calculated as a function of time. Furthermore, to analyze the interaction of the drug with the proteins, we performed RMSD of the drug during the course of the simulation, while minimizing the RMSD of the protein monomer/complex. In addition, to identify the protein residues interacting with the ligands, we calculated the average number of contacts made by the heavy atoms of the ligands with their protein counterparts defined by a 6 Å distance cutoff. We also calculated the occupancy of those interactions defined by the fraction of snapshots with at least one contact between the protein residues and the ligands. The production runs comprising 100 ns of simulation data were used for the interaction analysis.

#### 4.1.8. MM-PBSA Calculations

The average binding free energy is derived from the Gibbs free energy:

(1) $$G=E_{bnd}+E_{el}+E_{vdW}+G_{pol}+G_{apol}-TS$$

(2) $$G=G_{MM}+G_{pol}+G_{apol}-TS$$

where $G_{MM}$, $G_{pol}$ and $G_{apol}$ denote the molecular mechanics, polar and apolar contributions, respectively [45]. TS, the entropic contribution, was not included in our calculations. GROMACS g_mmpbsa package was used to obtain the $G_{MM}$, $G_{pol}$ and $G_{apol}$ contributions [46,47]. The package allows the user to define the protein and ligand during the calculation and measure the average binding free energy between the ligand and protein as follows:

(3) $$\Delta G=G^{complex}-\left(G^{protein}+G^{ligand}\right)$$

### 4.2. Experimental Methods

#### catL Activity Assay

rh catL was diluted to 40 µg/mL in an Assay Buffer (50 mM MES, 1 mM EDTA, 0.005% (*w*/*v*) Brij-35, pH 6.0). Diluted rh catL was incubated on ice for 15 min. The incubated 40 µg/mL rh catL was diluted to 40 ng/mL in Assay Buffer. An amount of 25 μL of catL inhibitor (SID 26681509) was loaded in 96-black-well transparent bottom plate; then, 25 μL of cathepsin L (1:1 dilution) was loaded and incubated at 37 °C for 30 min. The substrate was diluted to 80 µM in an Assay Buffer; then, 50 μL of substrate was added to each well and incubated as wrapped in foil at 37 °C for 1 h. Three replicates for each drug concentration were used. They were imaged on TECAN. All the tested drugs were diluted in DMSO to 2 mM (5 μL into 50 μL) and then added in amounts of 5 μL to each positive control well.

## Figures and Tables

**Table 1:** The list of ligands refined according to their toxicity, structural and functional similarity.

Drug Name	LD50 (mg/kg/day)	Function	Drug Name	LD50 (mg/kg/day)	Function
Sirolimus	2500	Anti-inflammatory	Elbasvir		Antimicrobial
Ciclesonide	2000		Cefpiramide	12,500	
Deflazacort	5200		Saquinavir	2500	
Dexamethasone M	873		Delamanid		
Icatibant	760		Bictegravir		
Triamcinolone	5000		Pibrentasvir		
Antrafenine	4000		Ritonavir		
Indacaterol	1600		Raltegravir		
Dihydroergotamine	8000	Migraine disorders	Drospirenone	2000	Reproductive health
Lasmiditan			Dutasteride	2000	
Ubrogepant	175		Estrone sulfate		
Valrubicin	109	Chemotherapeutic	Ursodeoxycholic acid		Natural hormones
Irinotecan	765		Oxytocin	514	
Paclitaxel			FAD		
Axitinib			Promacta		Blood disorder
Gleevec	120		Edoxaban		
Ibrutinib			Avatrombopag	160	
Capmatinib	2000		Gliquidone		Anti-diabetic
Vemurafenib			Glimepiride		
Digoxin	30	Cardiovascular disease	Troglitazone		
Candesartan cilexetil	2000		Vapsirol	500	Hyponatremia
Telmisartan			Tolvaptan		
Irbesartan			Plecanatide		Laxative
Nystatin	8000	Antimicrobial	Droperidol		Antipsychotic
Rifapentine	3300		Viibryd		
Rifaximin	2000		Aprepitant		
Simeprevir	1000		Mellaril-S		
Daclatasvir					

**Table 2:** The ligands and their targets obtained by MD simulations and ΔG calculations.

Protease–S Protein Complex		S Protein		Protease	
Target	Drug	Target	Drug	Target	Drug
catL-S1/S2’	Rifapentine	S1/S2	Capmatinib	catL	Rifapentine
	Digoxin		Avatrombopag		Drospirenone
	Nystatin		Cefpiramide		Digoxin
	Rifaximin		Rifapentine		Nystatin
trypsin-S1/S2	Drospirenone		Rifaximin	trypsin	Saquinavir
	Digoxin		Ciclesonide		Rifapentine
	Nystatin		Dihydroergotamine		Drospirenone
	Ubrogepant	S2’	Antrafenine		Digoxin
	Capmatinib		Irbesartan *	TMPRSS2	Saquinavir
TMPRSS2-S1/S2	Digoxin		Nebivolol *		Dexamethasone M
	Nystatin				Ubrogepant
	Dihydroergotamine				Nystatin
TMPRSS2-S2’	Ubrogepant				Digoxin
	Antrafenine				Rifapentine

* These ligands target D936Y variant of S2’.

**Table 3:** Cleavage sites on SARS-CoV and SARS-CoV-2 S proteins.

SARS-CoV S Protein	SARS-CoV-2 S Protein	Cleaved by
R667-S668	R685-S686 (S1/S2 boundary)	TMPRSS2 and trypsin
R797-S798	R815-S816 (S2’)	TMPRSS2 and trypsin
T678-M679	T696-M697 (S1/S2’ boundary)	catL

## Data Availability

Not applicable.

## Supplementary Materials

The following supporting information can be downloaded at: https://www.mdpi.com/article/10.3390/v14061129/s1, Figure S1: QMean scores and Ramachandran plots of modelled S protein and TMPRSS2 structures [48,49]; Figure S2: RMSD and RMSF plots for catL in the catL-drug complex simulations; Table S1: Top ~20 small molecule hits targeting the monomers obtained by in silico screening; Table S2: Top ~20 small molecule hits targeting the complexes obtained by in silico screening; Table S3: The average binding free energy of protein-ligand complexes; Table S4: Average number of contacts between residues of catL in catL-ligand interactions; Table S5: The occupancy percentage of catL-ligand interactions; Table S6: The grid parameters for all structures used in the docking; Table S7: Acute toxicity of drugs.
