# Supplementary material for: In Silico Screening and Testing of FDA-Approved Small Molecules to Block SARS-CoV-2 Entry to the Host Cell by Inhibiting Spike Protein Cleavage
Source: Viruses. 2022 May 24;14(6):1129. doi: 10.3390/v14061129 (PMC9231362; doi:10.3390/v14061129)
Supplement: Supplementary file 1 [file viruses-14-01129-s001.zip › viruses-1710365-supplementary.pdf]

# *Supplementary Materials*

## 1 Supplementary figures

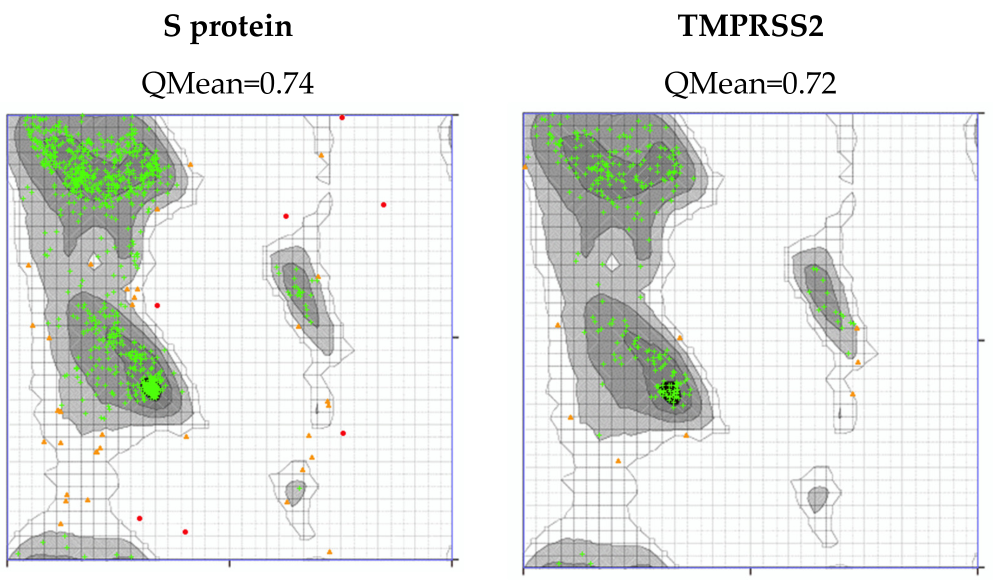


**Figure S1.** QMean scores[1] and Ramachandran plots[2] of modelled S protein and TMPRSS2 structures. 96% of the both S protein and TMPRSS2 models are classified as highly preferred observation (shown as green crosses), the remaining ~3% is classified as preferred observations (shown as orange triangles).

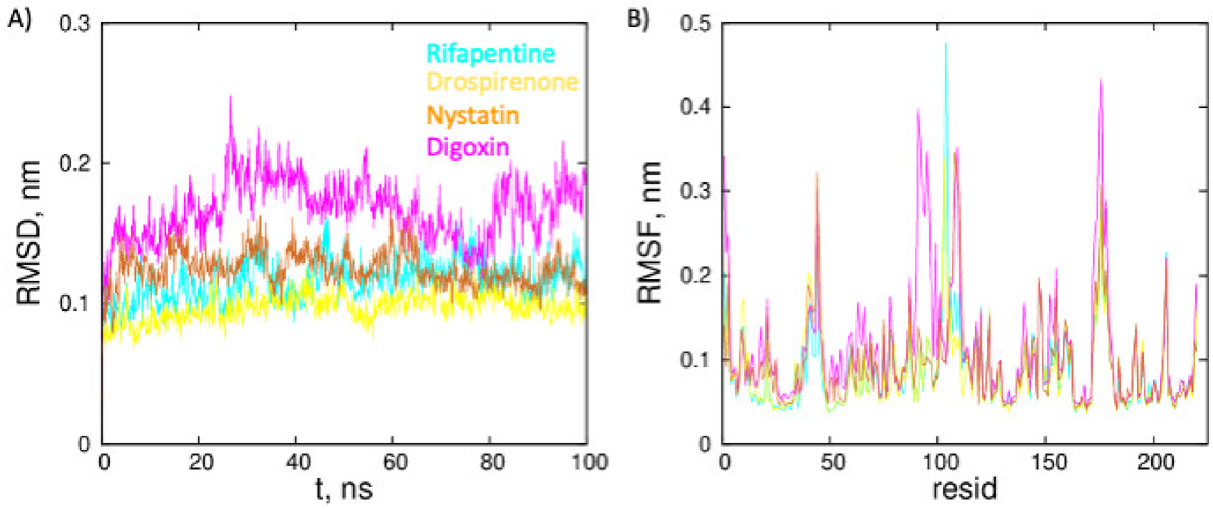


**Figure S2.** (A) RMSD and (B) RMSF plots for catL in the catL-drug complex simulations.

## 2 Supplementary tables

**Table S1.** Top ~20 small molecule hits targeting the monomers obtained by *in silico* screening *

| trypsin_1 | | | trypsin_2 | | | trypsin_3 | | |
|---|---|---|---|---|---|---|---|---|
| **Energy** | **Database** | **Name** | **Energy** | **Database** | **Name** | **Energy** | **Database** | **Name** |
| -12.1 | dbfda-world_494 | Nystatin | -12.6 | DrugBank_approved_980 | Candicidin | -13.6 | DrugBank_approved_980 | Candicidin |
| -10.5 | DrugBank_approved_980 | Candicidin | -11.3 | DrugBank_approved_1814 | Tannic acid | -11.9 | DrugBank_approved_2005 | Venetoclax |
| -10.1 | DrugBank_approved_1814 | Tannic acid | -10.8 | dbfda-world_1573 | Ecamsule | -11.2 | dbfda-world_531 | Amphotericin B |
| -9.9 | DrugBank_approved_1137 | Quinupristin | -10.7 | DrugBank_approved_2005 | Venetoclax | -11 | dbfda-world_495 | Nystatin |
| -9.9 | dbfda-world_1408 | Eribulin | -10.6 | dbfda-world_1138 | Solifenacin | -11 | dbfda-world_256 | Digoxin |
| -9.8 | dbfda-world_531 | Amphotericin B | -10.5 | dbfda-world_256 | Digoxin | -10.9 | TheBindingDB_780 | Juxtapid |
| -9.8 | DrugBank_approved_2005 | Venetoclax | -10.5 | dbfda-world_1519 | Trypan blue | -10.6 | DrugBank_approved_506 | Nystatin |
| -9.8 | dbfda-world_256 | Digoxin | -10.2 | DrugBank_approved_1597 | Canagliflozin | -10.5 | dbfda-world_496 | Nystatin |
| -9.7 | DrugBank_approved_2079 | Entrectinib | -10.2 | dbfda-world_1572 | Ecamsule | -10.5 | DrugBank_approved_1814 | Tannic acid |
| -9.7 | dbfda-world_495 | Nystatin | -10.2 | dbfda-world_545 | Ergotamine | -10.4 | DrugBank_approved_1409 | Eltrombopag |
| -9.7 | dbfda-world_496 | Nystatin | -10.2 | dbfda-world_494 | Nystatin | -10.4 | TheBindingDB_1035 | D.H.E. 45 |
| -9.7 | dbfda-world_736 | Sirolimus | -10.1 | dbfda-world_959 | Dutasteride | -10.4 | dbfda-world_545 | Ergotamine |
| -9.7 | TheBindingDB_332 | Tannic acid | -10.1 | DrugBank_approved_506 | Nystatin | -10.4 | TheBindingDB_559 | Epinephrine |
| -9.7 | dbfda-world_1028 | Rifapentine | -10.1 | dbfda-world_495 | Nystatin | -10.3 | dbfda-world_1534 | Paritaprevir |
| -9.6 | dbfda-world_252 | Valrubicin | -10.1 | DrugBank_approved_1232 | Rutin | -10.3 | TheBindingDB_1155 | Membraneblue |
| -9.6 | dbfda-world_1534 | Paritaprevir | -9.9 | TheBindingDB_568 | Vontrol | -10.3 | ChemBridge_26 | 7786009 |
| -9.6 | dbfda-world_176 | Dihydroergotamine | -9.9 | DrugBank_approved_555 | Ergotamine | -10.3 | DrugBank_approved_1565 | Lomitapide |
| -9.5 | TheBindingDB_939 | Oxytocin | -9.4 | DrugBank_approved_1057 | Saquinavir | -10.2 | DrugBank_approved_555 | Ergotamine |
| -9.5 | DrugBank_approved_506 | Nystatin | | | | -10.2 | TheBindingDB_955 | Tasigna |
| -9.5 | dbfda-world_493 | Nystatin | | | | -9.1 | DrugBank_approved_1057 | Saquinavir |

| catL_1 | | | catL_2 | | | catL_3 | | |
|---|---|---|---|---|---|---|---|---|
| **Energy** | **Database** | **Name** | **Energy** | **Database** | **Name** | **Energy** | **Database** | **Name** |
| -11.5 | dbfda-world_256 | Digoxin | -10.6 | dbfda-world_256 | Digoxin | -11.4 | dbfda-world_256 | Digoxin |
| -10.9 | DrugBank_approved_506 | Nystatin | -10.3 | DrugBank_approved_506 | Nystatin | -11.3 | DrugBank_approved_506 | Nystatin |
| -10.8 | dbfda-world_495 | Nystatin | -10 | dbfda-world_493 | Nystatin | -10.2 | dbfda-world_176 | Dihydroergotamine |
| -10.5 | DrugBank_approved_980 | Candicidin | -10 | DrugBank_approved_980 | Candicidin | -10.2 | dbfda-world_495 | Nystatin |
| -10.1 | dbfda-world_496 | Nystatin | -9.9 | DrugBank_approved_1137 | Quinupristin | -10 | DrugBank_approved_555 | Ergotamine |
| -10 | dbfda-world_1534 | Paritaprevir | -9.8 | dbfda-world_176 | Dihydroergotamine | -9.9 | dbfda-world_1299 | Midostaurin |
| -10 | dbfda-world_730 | Conivaptan | -9.7 | dbfda-world_496 | Nystatin | -9.8 | DrugBank_approved_1137 | Quinupristin |
| -9.9 | dbfda-world_176 | Dihydroergotamine | -9.3 | TheBindingDB_821 | Camptosar | -9.8 | dbfda-world_531 | Amphotericin B |
| -9.6 | DrugBank_approved_1137 | Quinupristin | -9.3 | DrugBank_approved_540 | Amphotericin B | -9.7 | TheBindingDB_821 | Camptosar |
| -9.6 | dbfda-world_1519 | Trypan blue | -9.2 | dbfda-world_1048 | Rifaximin | -9.7 | DrugBank_approved_3 | Desmopressin |
| -9.5 | DrugBank_approved_2079 | Entrectinib | -9.2 | dbfda-world_549 | Eplerenone | -9.7 | dbfda-world_496 | Nystatin |
| -9.4 | DrugBank_approved_555 | Ergotamine | -9.2 | dbfda-world_545 | Ergotamine | -9.7 | dbfda-world_545 | Ergotamine |
| -9.4 | dbfda-world_456 | Ivermectin | -9.2 | TheBindingDB_559 | Epinephrine | -9.7 | dbfda-world_494 | Nystatin |
| -9.4 | dbfda-world_531 | Amphotericin B | -9.2 | dbfda-world_1519 | Trypan blue | -9.6 | dbfda-world_1534 | Paritaprevir |
| -9.4 | DrugBank_approved_540 | Amphotericin B | -9.2 | dbfda-world_494 | Nystatin | -9.6 | dbfda-world_456 | Ivermectin |
| -9.3 | TheBindingDB_821 | Camptosar | -9.1 | dbfda-world_1534 | Paritaprevir | -9.5 | dbfda-world_1629 | Naldemedine |
| -9.3 | dbfda-world_1628 | Velpatasvir | -9.1 | dbfda-world_495 | Nystatin | -9.5 | DrugBank_approved_1962 | Bisoctrizole |
| -9.2 | dbfda-world_545 | Ergotamine | -9.1 | DrugBank_approved_3 | Desmopressin | -9.5 | TheBindingDB_559 | B.H And Epinephrine |
| -9.2 | dbfda-world_1028 | Rifapentine | -9.1 | dbfda-world_531 | Amphotericin B | -9.4 | DrugBank_approved_90 | Adapalene |
| | | | -9.1 | ChemBridge_26 | 7786009 | -9.4 | TheBindingDB_938 | Concentraid |

| TMPRSS2_1 | | | TMPRSS2_2 | | | TMPRSS2_3 | | |
|---|---|---|---|---|---|---|---|---|
| **Energy** | **Database** | **Name** | **Energy** | **Database** | **Name** | **Energy** | **Database** | **Name** |
| -11.2 | DrugBank_approved_2108 | Cantharidin | -11.8 | DrugBank_approved_980 | Candicidin | -11.3 | DrugBank_approved_2005 | Venetoclax |
| -10.7 | dbfda-world_256 | Digoxin | -10.7 | DrugBank_approved_2108 | Cantharidin | -10.9 | DrugBank_approved_1137 | Quinupristin |
| -10.1 | DrugBank_approved_1137 | Quinupristin | -10.2 | DrugBank_approved_811 | Dactinomycin | -10.7 | DrugBank_approved_980 | Candicidin |
| -9.7 | DrugBank_approved_1401 | Icatibant | -10.2 | DrugBank_approved_2005 | Venetoclax | -10.4 | dbfda-world_176 | Dihydroergotamine |

| | | | | | | | | |
|---|---|---|---|---|---|---|---|---|
| -9.6 | DrugBank_approved_506 | Nystatin | -10.1 | dbfda-world_256 | Digoxin | -10.3 | DrugBank_approved_1401 | Icatibant |
| -9.6 | DrugBank_approved_980 | Candicidin | -10 | DrugBank_approved_486 | Bacitracin | -10.3 | dbfda-world_531 | Amphotericin B |
| -9.5 | dbfda-world_531 | Amphotericin B | -9.8 | dbfda-world_494 | Nystatin | -10.3 | DrugBank_approved_2108 | Cantharidin |
| -9.4 | DrugBank_approved_555 | Ergotamine | -9.7 | DrugBank_approved_1137 | Quinupristin | -10.3 | dbfda-world_494 | Nystatin |
| -9.4 | dbfda-world_496 | Nystatin | -9.6 | DrugBank_approved_1339 | Nilotinib | -10.1 | DrugBank_approved_506 | Nystatin |
| -9.4 | dbfda-world_1263 | Eltrombopag | -9.4 | DrugBank_approved_506 | Nystatin | -10.1 | DrugBank_approved_377 | Acetyldigitoxin |
| -9.4 | dbfda-world_1519 | Trypan blue | -9.4 | dbfda-world_531 | Amphotericin B | -10 | DrugBank_approved_2441 | Ubrogepant |
| -9.3 | DrugBank_approved_2441 | Ubrogepant | -9.4 | DrugBank_approved_1639 | Ledipasvir | -10 | dbfda-world_1299 | Midostaurin |
| -9.3 | DrugBank_approved_955 | Dutasteride | -9.3 | TheBindingDB_955 | Tasigna | -10 | dbfda-world_256 | Digoxin |
| -9.2 | dbfda-world_493 | Nystatin | -9.3 | DrugBank_approved_377 | Acetyldigitoxin | -9.9 | dbfda-world_1534 | Paritaprevir |
| -9.2 | DrugBank_approved_1814 | Tannic acid | -9.3 | TheBindingDB_910 | Avagard | -9.9 | DrugBank_approved_3 | Desmopressin |
| -9.2 | DrugBank_approved_2179 | Plecanatide | -9.3 | TheBindingDB_1061 | Vaprisol | -9.9 | dbfda-world_1519 | Trypan blue |
| -9.1 | DrugBank_approved_2429 | Dexamethasone M | -9.2 | dbfda-world_735 | Sirolimus | -9.8 | DrugBank_approved_2429 | Dexamethasone M |
| -9.1 | dbfda-world_494 | Nystatin | -9.2 | dbfda-world_496 | Nystatin | -9.8 | dbfda-world_496 | Nystatin |
| -8.8 | DrugBank_approved_1057 | Saquinavir | -9.1 | dbfda-world_1534 | Paritaprevir | -9.8 | DrugBank_approved_1814 | Tannic acid |
| | | | -9.1 | TheBindingDB_1155 | Membraneblue | | | |

| S1/S2_1 | | | S1/S2_2 | | | S1/S2_3 | | |
|---|---|---|---|---|---|---|---|---|
| **Energy** | **Database** | **Name** | **Energy** | **Database** | **Name** | **Energy** | **Database** | **Name** |
| -11.7 | DrugBank_approved_980 | Candicidin | -11.1 | DrugBank_approved_980 | Candicidin | -9.2 | dbfda-world_495 | Nystatin |
| -10.7 | dbfda-world_494 | Nystatin | -10.1 | dbfda-world_1519 | Trypan blue | -9 | DrugBank_approved_980 | Candicidin |
| -10.5 | dbfda-world_176 | Dihydroergotamine | -9.5 | dbfda-world_256 | Digoxin | -9 | dbfda-world_494 | Nystatin |
| -10.4 | DrugBank_approved_3 | Desmopressin | -9.3 | dbfda-world_1048 | Rifaximin | -8.9 | dbfda-world_256 | Digoxin |
| -10.3 | TheBindingDB_1156 | Evans Blue | -9.2 | DrugBank_approved_2441 | Ubrogepant | -8.7 | DrugBank_approved_555 | Ergotamine |
| -10.2 | DrugBank_approved_2441 | Ubrogepant | -9.2 | dbfda-world_494 | Nystatin | -8.5 | TheBindingDB_1061 | Vaprisol |
| -10.1 | DrugBank_approved_555 | Ergotamine | -9.1 | DrugBank_approved_8 | Abarelix | -8.5 | dbfda-world_1519 | Trypan blue |
| -10.1 | dbfda-world_256 | Digoxin | -9 | dbfda-world_1627 | Lifitegrast | -8.4 | DrugBank_approved_2079 | Entrectinib |
| -10 | DrugBank_approved_1639 | Ledipasvir | -9 | TheBindingDB_625 | Cafergot | -8.4 | DrugBank_approved_1814 | Tannic acid |
| -9.9 | DrugBank_approved_1814 | Tannic acid | -9 | DrugBank_approved_2108 | Cantharidin | -8.4 | DrugBank_approved_2005 | Venetoclax |
| -9.9 | TheBindingDB_1061 | Vaprisol | -8.8 | dbfda-world_690 | Natamycin | -8.3 | DrugBank_approved_506 | Nystatin |
| -9.9 | DrugBank_approved_2005 | Venetoclax | -8.7 | dbfda-world_736 | Sirolimus | -8.3 | DrugBank_approved_2056 | Capmatinib |
| -9.8 | DrugBank_approved_2082 | Avatrombopag | -8.7 | dbfda-world_1117 | Ciclesonide | -8.3 | dbfda-world_493 | Nystatin |
| -9.8 | DrugBank_approved_1736 | Trypan blue | -8.7 | DrugBank_approved_618 | Irinotecan | -8.3 | dbfda-world_496 | Nystatin |
| -9.8 | dbfda-world_531 | Amphotericin B | -8.6 | DrugBank_approved_1137 | Quinupristin | -8.3 | dbfda-world_531 | Amphotericin B |
| -9.8 | DrugBank_approved_479 | Imatinib | -8.6 | dbfda-world_176 | Dihydroergotamine | -8.3 | TheBindingDB_770 | Eptifibatide |
| -9.7 | dbfda-world_1283 | Simeprevir | -8.6 | dbfda-world_496 | Nystatin | -8.3 | DrugBank_approved_486 | Bacitracin |
| -9.7 | DrugBank_approved_506 | Nystatin | -8.6 | DrugBank_approved_540 | Amphotericin B | -8.2 | TheBindingDB_230 | Votrient |
| -9.7 | dbfda-world_1534 | Paritaprevir | -8.6 | DrugBank_approved_302 | Cefpiramide | -8.2 | dbfda-world_730 | Conivaptan |
| -9.7 | dbfda-world_495 | Nystatin | -8.6 | DrugBank_approved_1639 | Ledipasvir | | | |

| S1/S2'_1$^{+}$ | | | S1/S2'_2$^{+}$ | | | S1/S2'_3$^{+}$ | | |
|---|---|---|---|---|---|---|---|---|
| **Energy** | **Database** | **Name** | **Energy** | **Database** | **Name** | **Energy** | **Database** | **Name** |
| -8.5 | DrugBank_approved_2108 | Cantharidin | -9 | dbfda-world_256 | Digoxin | -8.9 | dbfda-world_494 | Nystatin |
| -8.5 | dbfda-world_494 | Nystatin | -8.4 | dbfda-world_176 | Dihydroergotamine | -8.7 | dbfda-world_256 | Digoxin |
| -8.4 | dbfda-world_495 | Nystatin | -8.4 | dbfda-world_496 | Nystatin | -8.6 | DrugBank_approved_540 | Amphotericin B |
| -8.4 | DrugBank_approved_980 | Candicidin | -8.2 | DrugBank_approved_980 | Candicidin | -8.6 | DrugBank_approved_2108 | Cantharidin |
| -8.3 | dbfda-world_689 | Natamycin | -8 | dbfda-world_690 | Natamycin | -8.5 | DrugBank_approved_980 | Candicidin |
| -8.3 | dbfda-world_256 | Digoxin | -7.9 | dbfda-world_1407 | Eribulin | -8.2 | DrugBank_approved_3 | Desmopressin |
| -8.2 | dbfda-world_690 | Natamycin | -7.9 | dbfda-world_545 | Ergotamine | -8.2 | dbfda-world_736 | Sirolimus |
| -8.1 | dbfda-world_493 | Nystatin | -7.9 | dbfda-world_1519 | Trypan blue | -8.2 | DrugBank_approved_2005 | Venetoclax |
| -8.1 | dbfda-world_1519 | Trypan blue | -7.8 | dbfda-world_1028 | Rifapentine | -8.1 | dbfda-world_1048 | Rifaximin |
| -7.8 | DrugBank_approved_506 | Nystatin | -7.7 | DrugBank_approved_2056 | Capmatinib | -8 | DrugBank_approved_1137 | Quinupristin |
| -7.8 | dbfda-world_736 | Sirolimus | -7.7 | dbfda-world_494 | Nystatin | -8 | dbfda-world_495 | Nystatin |
| -7.8 | dbfda-world_531 | Amphotericin B | -7.6 | DrugBank_approved_2079 | Entrectinib | -7.9 | dbfda-world_1282 | Simeprevir |
| -7.7 | DrugBank_approved_1137 | Quinupristin | -7.6 | DrugBank_approved_555 | Ergotamine | -7.9 | dbfda-world_496 | Nystatin |
| -7.7 | dbfda-world_496 | Nystatin | -7.6 | dbfda-world_1627 | Lifitegrast | -7.9 | TheBindingDB_660 | Ivacaftor |
| -7.6 | dbfda-world_1283 | Simeprevir | -7.6 | dbfda-world_531 | Amphotericin B | -7.8 | DrugBank_approved_506 | Nystatin |
| -7.6 | DrugBank_approved_2005 | Venetoclax | -7.6 | TheBindingDB_770 | Eptifibatide | -7.8 | dbfda-world_689 | Natamycin |
| -7.6 | dbfda-world_545 | Ergotamine | -7.6 | DrugBank_approved_2005 | Venetoclax | -7.8 | dbfda-world_531 | Amphotericin B |

| | | | | | | | | |
|---|---|---|---|---|---|---|---|---|
| -7.5 | dbfda-world_1573 | Ecamsule | -7.5 | TheBindingDB_1035 | D.H.E. 45 | -7.8 | dbfda-world_545 | Ergotamine |
| | | | -7.4 | dbfda-world_495 | Nystatin | -7.7 | dbfda-world_1629 | Naldemedine |
| | | | -7.4 | DrugBank_approved_2441 | Ubrogepant | -7.7 | DrugBank_approved_555 | Ergotamine |

| S2' | | | S2'_D936Y | | |
|---|---|---|---|---|---|
| **Energy** | **Database** | **Name** | **Energy** | **Database** | **Name** |
| -12.8 | DrugBank_approved_980 | Candicidin | -10.4 | DrugBank_approved_1137 | Quinupristin |
| -12.6 | dbfda-world_256 | Digoxin | -10.3 | dbfda-world_256 | Digoxin |
| -11.6 | DrugBank_approved_2005 | Venetoclax | -10.2 | DrugBank_approved_955 | Dutasteride |
| -11.4 | DrugBank_approved_2108 | Cantharidin | -10.1 | TheBindingDB_722 | Viracept |
| -11.2 | DrugBank_approved_1162 | Antrafenine | -10.1 | dbfda-world_953 | Atovaquone |
| -11 | TheBindingDB_780 | Juxtapid | -9.9 | dbfda-world_1477 | Olaparib |
| -10.9 | dbfda-world_1629 | Naldemedine | -9.9 | dbfda-world_1070 | Docetaxel |
| -10.9 | TheBindingDB_1285 | IC-Green | -9.8 | TheBindingDB_952 | Glyxambi |
| -10.8 | DrugBank_approved_377 | Acetyldigitoxin | -9.8 | dbfda-world_1282 | Simeprevir |
| -10.8 | DrugBank_approved_1565 | Lomitapide | -9.8 | TheBindingDB_689 | Natamycin |
| -10.7 | DrugBank_approved_1137 | Quinupristin | -9.8 | dbfda-world_1179 | Deoxycholic acid |
| -10.7 | TheBindingDB_408 | Vaprisol | -9.8 | TheBindingDB_1061 | Vaprisol |
| -10.6 | DrugBank_approved_1401 | Icatibant | -9.8 | DrugBank_approved_720 | Conivaptan |
| -10.6 | dbfda-world_1519 | Trypan blue | -9.8 | - | Drospirenone |
| -10.5 | DrugBank_approved_8 | Abarelix | -9.7 | DrugBank_approved_868 | Irbesartan |
| -10.3 | TheBindingDB_821 | Camptosar | -9.7 | DrugBank_approved_3 | Desmopressin |
| -10.3 | dbfda-world_656 | Candesartan cilexetil | -9.7 | DrugBank_approved_2058 | Bictegravir |
| -10.3 | dbfda-world_1081 | Posaconazole | -9.7 | dbfda-world_1058 | Saquinavir |
| | | | -9.7 | TheBindingDB_688 | Irbesartan |

*Unit of energy is kcal/mol. AutoDock Vina (version 1.1.2, Linux) was used for the docking.
+ S1/S2′ corresponds to the cleavage site targeted by catL (T696-M697) on S1/S2 boundary.

**Table S2.** Top ~20 small molecule hits targeting the complexes obtained by *in silico* screening *

| trypsin_1-S1/S2_1 | | | trypsin_1-S1/S2_2 | | | trypsin_2-S1/S2_1 | | |
|---|---|---|---|---|---|---|---|---|
| **Energy** | **Database** | **Name** | **Energy** | **Database** | **Name** | **Energy** | **Database** | **Name** |
| -11.7 | dbfda-world_1263 | Eltrombopag | -11.5 | DrugBank_approved_555 | Ergotamine | -12.2 | dbfda-world_1629 | Naldemedine |
| -11.5 | DrugBank_approved_1773 | Lumacaftor | -11.4 | dbfda-world_1654 | Glecaprevir | -11.4 | DrugBank_approved_1401 | Icatibant |
| -11.4 | TheBindingDB_253 | Lapatinib | -11.4 | dbfda-world_1629 | Naldemedine | -11.2 | DrugBank_approved_2005 | Venetoclax |
| -11.3 | ChemBridge_26 | 7786009 | -11.4 | dbfda-world_256 | Digoxin | -11.1 | DrugBank_approved_3 | Desmopressin |
| -11.3 | dbfda-world_1572 | Ecamsule | -11.1 | dbfda-world_690 | Natamycin | -11.1 | DrugBank_approved_980 | Candicidin |
| -11.3 | DrugBank_approved_2155 | Pexidartinib | -11 | dbfda-world_1075 | Retapamulin | -11 | dbfda-world_1519 | Trypan blue |
| -11.2 | TheBindingDB_955 | Tasigna | -11 | DrugBank_approved_2056 | Capmatinib | -10.9 | dbfda-world_496 | Nystatin |
| -11 | TheBindingDB_565 | Nupercaine | -11 | dbfda-world_1070 | Docetaxel | -10.8 | DrugBank_approved_2066 | Revefenacin |
| -10.9 | DrugBank_approved_1162 | Antrafenine | -11 | dbfda-world_1028 | Rifapentine | -10.8 | dbfda-world_256 | Digoxin |
| -10.9 | DrugBank_approved_2441 | Ubrogepant | -10.9 | dbfda-world_176 | Dihydroergotamine | -10.7 | DrugBank_approved_259 | Valrubicin |
| -10.9 | DrugBank_approved_2056 | Capmatinib | -10.9 | dbfda-world_689 | Natamycin | -10.7 | DrugBank_approved_1263 | FAD |
| -10.9 | DrugBank_approved_1339 | Nilotinib | -10.9 | TheBindingDB_559 | Epinephrine | -10.6 | TheBindingDB_955 | Tasigna |
| -10.9 | TheBindingDB_529 | Thyroid Hormone | -10.9 | DrugBank_approved_980 | Candicidin | -10.5 | dbfda-world_176 | Dihydroergotamine |
| -10.9 | DrugBank_approved_1442 | Pazopanib | -10.8 | dbfda-world_545 | Ergotamine | -10.5 | DrugBank_approved_1339 | Nilotinib |
| -10.8 | DrugBank_approved_2005 | Venetoclax | -10.7 | dbfda-world_1534 | Paritaprevir | -10.5 | dbfda-world_730 | Conivaptan |
| -10.8 | TheBindingDB_572 | Droperidol | -10.7 | TheBindingDB_625 | Cafergot | -10.5 | DrugBank_approved_1814 | Tannic acid |
| -10.7 | DrugBank_approved_1652 | Ibrutinib | -10.7 | dbfda-world_1519 | Trypan blue | -10.5 | dbfda-world_1428 | Ponatinib |
| -10.7 | DrugBank_approved_1814 | Tannic Acid | -10.6 | dbfda-world_1621 | Venetoclax | -10.5 | DrugBank_approved_1632 | Diosmin |
| -10.7 | DrugBank_approved_2058 | Bictegravir | -10.6 | dbfda-world_456 | Ivermectin | -10.5 | dbfda-world_545 | Ergotamine |
| -10.7 | ChemBridge_29 | 7928214 | -10.6 | dbfda-world_1074 | Retapamulin | -10.3 | dbfda-world_1217 | Nilotinib |
| -10.7 | DrugBank_approved_1543 | Triptorelin | -10.5 | DrugBank_approved_377 | Acetyldigitoxin | -10.3 | TheBindingDB_232 | Gleevec |
| -10.7 | TheBindingDB_780 | Juxtapid | -10.5 | - | Camostat | -10.3 | DrugBank_approved_2108 | Cantharidin |
| -9.5 | - | Camostat | | | | | | |

| trypsin_1-S1/S2_2 | | | trypsin_2-S1/S2_2 | | | trypsin_3-S1/S2_2 | | |
|---|---|---|---|---|---|---|---|---|
| **Energy** | **Database** | **Name** | **Energy** | **Database** | **Name** | **Energy** | **Database** | **Name** |
| -12.2 | DrugBank_approved_980 | Candicidin | -11.3 | DrugBank_approved_1137 | Quinupristin | -11 | DrugBank_approved_980 | Candicidin |
| -11.6 | dbfda-world_494 | Nystatin | -11.3 | DrugBank_approved_980 | Candicidin | -10.7 | dbfda-world_256 | Digoxin |
| -10.9 | dbfda-world_256 | Digoxin | -11.2 | dbfda-world_256 | Digoxin | -10.5 | dbfda-world_496 | Nystatin |
| -10.4 | dbfda-world_493 | Nystatin | -11 | dbfda-world_176 | Dihydroergotamine | -10.4 | dbfda-world_495 | Nystatin |
| -10.4 | DrugBank_approved_2108 | Cantharidin | -11 | DrugBank_approved_1814 | Tannic acid | -10.4 | DrugBank_approved_2108 | Cantharidin |
| -10.3 | DrugBank_approved_506 | Nystatin | -10.9 | DrugBank_approved_720 | Conivaptan | -10.3 | dbfda-world_1519 | Trypan blue |
| -9.9 | DrugBank_approved_3 | Desmopressin | -10.8 | dbfda-world_1621 | Venetoclax | -10.3 | dbfda-world_494 | Nystatin |
| -9.9 | dbfda-world_531 | Amphotericin B | -10.8 | TheBindingDB_955 | Tasigna | -10.1 | DrugBank_approved_506 | Nystatin |
| -9.8 | dbfda-world_176 | Dihydroergotamine | -10.8 | dbfda-world_1534 | Paritaprevir | -10 | dbfda-world_1534 | Paritaprevir |
| -9.8 | dbfda-world_496 | Nystatin | -10.8 | DrugBank_approved_1339 | Nilotinib | -9.7 | dbfda-world_493 | Nystatin |
| -9.8 | DrugBank_approved_1401 | Icatibant | -10.8 | ChemBridge_26 | 7786009 | -9.7 | DrugBank_approved_486 | Bacitracin |
| -9.8 | dbfda-world_1519 | Trypan blue | -10.7 | DrugBank_approved_2005 | Venetoclax | -9.6 | dbfda-world_1299 | Midostaurin |
| -9.6 | DrugBank_approved_1137 | Quinupristin | -10.6 | dbfda-world_1217 | Nilotinib | -9.6 | dbfda-world_531 | Amphotericin B |
| -9.6 | dbfda-world_1534 | Paritaprevir | -10.6 | TheBindingDB_71 | Ritonavir | -9.5 | dbfda-world_176 | Dihydroergotamine |
| -9.6 | dbfda-world_495 | Nystatin | -10.5 | dbfda-world_75 | Adapalene | -9.4 | dbfda-world_1654 | Glecaprevir |
| -9.6 | DrugBank_approved_486 | Bacitracin | -10.4 | TheBindingDB_568 | Vontrol | -9.4 | dbfda-world_1477 | Olaparib |
| -9.5 | DrugBank_approved_5 | Daptomycin | -10.4 | ChemBridge_10 | 7793889 | -9.4 | TheBindingDB_299 | Dactinomycin |
| -9.5 | dbfda-world_1028 | Rifapentine | -10.3 | DrugBank_approved_2079 | Entrectinib | -9.4 | TheBindingDB_1320 | Tannic Acid |
| -9.4 | DrugBank_approved_1028 | Rifapentine | -10.3 | TheBindingDB_1229 | Vemurafenib | -9.4 | TheBindingDB_278 | Rapamune |
| -9.3 | DrugBank_approved_540 | Amphotericin B | -10.3 | DrugBank_approved_506 | Nystatin | -9.3 | dbfda-world_1283 | Simeprevir |
| -9.3 | DrugBank_approved_2021 | Velpatasvir | -10.3 | DrugBank_approved_555 | Ergotamine | -9.3 | dbfda-world_1455 | Ledipasvir |
| -9.2 | DrugBank_approved_1814 | Tannic acid | | | | -9.3 | TheBindingDB_625 | Cafergot |

| trypsin_1-S2' | | | trypsin_2-S2' | | | trypsin_3-S2' | | |
|---|---|---|---|---|---|---|---|---|
| **Energy** | **Database** | **Name** | **Energy** | **Database** | **Name** | **Energy** | **Database** | **Name** |
| -11.4 | DrugBank_approved_2108 | Cantharidin | -12.8 | DrugBank_approved_2108 | Cantharidin | -11.7 | DrugBank_approved_1137 | Quinupristin |
| -11.4 | dbfda-world_494 | Nystatin | -11 | DrugBank_approved_506 | Nystatin | -11.6 | DrugBank_approved_980 | Candicidin |
| -11.2 | TheBindingDB_760 | Prelay | -10.6 | DrugBank_approved_980 | Candicidin | -11 | dbfda-world_256 | Digoxin |
| -11.1 | DrugBank_approved_1162 | Antrafenine | -10.2 | dbfda-world_256 | Digoxin | -10.9 | dbfda-world_1572 | Ecamsule |
| -11 | TheBindingDB_955 | Tasigna | -10.2 | dbfda-world_494 | Nystatin | -10.5 | DrugBank_approved_506 | Nystatin |
| -11 | TheBindingDB_110 | Alimta | -10.1 | DrugBank_approved_3 | Desmopressin | -10.4 | TheBindingDB_1061 | Vapsirol |
| -10.9 | DrugBank_approved_2045 | Lasmiditan | -10 | dbfda-world_1654 | Glecaprevir | -10.2 | dbfda-world_1621 | Venetoclax |
| -10.9 | DrugBank_approved_2074 | Duvelisib | -9.9 | DrugBank_approved_555 | Ergotamine | -10.2 | DrugBank_approved_1773 | Lumacaftor |
| -10.8 | DrugBank_approved_946 | Atovaquone | -9.8 | DrugBank_approved_1137 | Quinupristin | -10.1 | DrugBank_approved_1814 | Tannic acid |
| -10.7 | ChemBridge_25 | 7931205 | -9.8 | TheBindingDB_278 | Rapamune | -10.1 | DrugBank_approved_1639 | Ledipasvir |
| -10.7 | dbfda-world_1654 | Glecaprevir | -9.8 | TheBindingDB_873 | Viibryd | -10.1 | DrugBank_approved_2005 | Venetoclax |
| -10.7 | DrugBank_approved_1814 | Tannic acid | -9.7 | dbfda-world_495 | Nystatin | -10 | TheBindingDB_938 | Concentraid |
| -10.7 | dbfda-world_256 | Digoxin | -9.7 | DrugBank_approved_2002 | Elbasvir | -10 | DrugBank_approved_555 | Ergotamine |
| -10.7 | dbfda-world_1519 | Trypan blue | -9.7 | dbfda-world_1192 | Estrone sulfate | -10 | dbfda-world_493 | Nystatin |
| -10.7 | DrugBank_approved_101 | Glimepiride | -9.7 | TheBindingDB_174 | Raltegravir | -10 | DrugBank_approved_486 | Bacitracin |
| -10.6 | DrugBank_approved_2079 | Entrectinib | -9.7 | dbfda-world_1519 | Trypan blue | -9.9 | dbfda-world_496 | Nystatin |
| -10.6 | dbfda-world_496 | Nystatin | -9.6 | DrugBank_approved_955 | Dutasteride | -9.9 | TheBindingDB_947 | Aprepitant |
| -10.5 | dbfda-world_495 | Nystatin | -9.6 | dbfda-world_1306 | Axitinib | -9.9 | DrugBank_approved_720 | Conivaptan |
| -10.5 | ChemBridge_37 | 7927879 | -9.6 | TheBindingDB_1061 | Vapsirol | -9.9 | dbfda-world_494 | Nystatin |
| -10.4 | dbfda-world_176 | Dihydroergotamine | -9.6 | dbfda-world_1133 | Ursodeoxycholic A. | -9.8 | dbfda-world_1534 | Paritaprevir |

| trypsin_1-S2'_D936Y | | | trypsin_2-S2'_D936Y | | | trypsin_3-S2'_D936Y | | |
|---|---|---|---|---|---|---|---|---|
| **Energy** | **Database** | **Name** | **Energy** | **Database** | **Name** | **Energy** | **Database** | **Name** |

| | | | | | | | | |
|---|---|---|---|---|---|---|---|---|
| -12.2 | DrugBank_approved_980 | Candicidin | -12 | dbfda-world_1572 | Ecamsule | -12.9 | DrugBank_approved_980 | Candicidin |
| -11.4 | DrugBank_approved_1814 | Tannic acid | -11.7 | dbfda-world_494 | Nystatin | -12.6 | dbfda-world_256 | Digoxin |
| -11.4 | DrugBank_approved_2108 | Cantharidin | -11.3 | DrugBank_approved_980 | Candicidin | -12.3 | DrugBank_approved_1137 | Quinupristin |
| -10.9 | ChemBridge_26 | 7786009 | -11.1 | DrugBank_approved_506 | Nystatin | -11.6 | DrugBank_approved_2005 | Venetoclax |
| -10.8 | TheBindingDB_568 | Vontrol | -11.1 | dbfda-world_730 | Conivaptan | -11.6 | dbfda-world_1519 | Trypan blue |
| -10.8 | dbfda-world_495 | Nystatin | -11 | TheBindingDB_695 | Adapalene | -11.5 | dbfda-world_176 | Dihydroergotamine |
| -10.8 | DrugBank_approved_1776 | Rolapitant | -10.9 | dbfda-world_1085 | Paliperidone | -11.5 | dbfda-world_493 | Nystatin |
| -10.8 | dbfda-world_256 | Digoxin | -10.9 | DrugBank_approved_8 | Abarelix | -11.5 | DrugBank_approved_8 | Abarelix |
| -10.6 | DrugBank_approved_532 | Aprepitant | -10.8 | DrugBank_approved_125 | Ziprasidone | -11.4 | DrugBank_approved_2108 | Cantharidin |
| -10.6 | DrugBank_approved_1137 | Quinupristin | -10.8 | dbfda-world_496 | Nystatin | -11.2 | DrugBank_approved_555 | Ergotamine |
| -10.5 | DrugBank_approved_3 | Desmopressin | -10.8 | dbfda-world_256 | Digoxin | -11.2 | dbfda-world_495 | Nystatin |
| -10.5 | DrugBank_approved_1361 | Indacaterol | -10.7 | dbfda-world_1627 | Lifitegrast | -11.2 | dbfda-world_1282 | Simeprevir |
| -10.5 | DrugBank_approved_2005 | Venetoclax | -10.7 | dbfda-world_1084 | Paliperidone | -11.2 | TheBindingDB_1320 | Tannic Acid |
| -10.4 | DrugBank_approved_506 | Nystatin | -10.6 | dbfda-world_22 | Ergocalciferol | -11.1 | dbfda-world_531 | Amphotericin B |
| -10.4 | DrugBank_approved_1409 | Eltrombopag | -10.6 | DrugBank_approved_590 | Risperidone | -11.0 | dbfda-world_1075 | Retapamulin |
| -10.4 | DrugBank_approved_2056 | Capmatinib | -10.6 | TheBindingDB_989 | Promacta | -10.9 | dbfda-world_1629 | Velpatasvir |
| -10.4 | dbfda-world_496 | Nystatin | -10.6 | TheBindingDB_174 | Raltegravir | -10.9 | dbfda-world_1534 | Paritaprevir |
| -10.4 | DrugBank_approved_1669 | Olaparib | -10.5 | dbfda-world_495 | Nystatin | -10.9 | DrugBank_approved_377 | Acetyldigitoxin |
| -10.3 | dbfda-world_1212 | Nebivolol | -10.5 | dbfda-world_954 | Atovaquone | -10.8 | dbfda-world_730 | Conivaptan |
| -10.3 | DrugBank_approved_2274 | Netarsudil | | | | -10.8 | TheBindingDB_332 | Tannic acid |

| TMPRSS2_1-S1/S2_1 | | | TMPRSS2_2-S1/S2_1 | | |
|---|---|---|---|---|---|
| **Energy** | **Database** | **Name** | **Energy** | **Database** | **Name** |
| -13.6 | dbfda-world_256 | Digoxin | -11.7 | dbfda-world_496 | Nystatin |
| -13.2 | dbfda-world_531 | Amphotericin B | -11.6 | dbfda-world_256 | Digoxin |
| -12.8 | DrugBank_approved_1137 | Quinupristin | -11.1 | dbfda-world_1534 | Paritaprevir |
| -12.6 | TheBindingDB_299 | Dactinomycin | -11.1 | TheBindingDB_332 | Tannic acid |
| -12.5 | dbfda-world_493 | Nystatin | -11 | DrugBank_approved_526 | Nafarelin |
| -12.5 | dbfda-world_496 | Nystatin | -10.9 | DrugBank_approved_377 | Acetyldigitoxin |
| -12.4 | DrugBank_approved_811 | Dactinomycin | -10.9 | DrugBank_approved_2005 | Venetoclax |
| -12.4 | dbfda-world_494 | Nystatin | -10.8 | dbfda-world_809 | Telmisartan |
| -12.1 | dbfda-world_495 | Nystatin | -10.8 | DrugBank_approved_1543 | Triptorelin |
| -12 | DrugBank_approved_506 | Nystatin | -10.8 | DrugBank_approved_980 | Candicidin |
| -12 | DrugBank_approved_1814 | Tannic acid | -10.7 | TheBindingDB_938 | Concentraid |
| -12 | DrugBank_approved_980 | Candicidin | -10.7 | DrugBank_approved_555 | Ergotamine |
| -11.8 | dbfda-world_1519 | Trypan blue | -10.7 | dbfda-world_176 | Dihydroergotamine |
| -11.6 | DrugBank_approved_2005 | Venetoclax | -10.7 | dbfda-world_1281 | Simeprevir |
| -11.6 | dbfda-world_545 | Ergotamine | -10.7 | dbfda-world_494 | Nystatin |
| -11.4 | dbfda-world_1534 | Paritaprevir | -10.6 | dbfda-world_1299 | Midostaurin |
| -11.4 | TheBindingDB_1156 | Evans Blue | -10.6 | DrugBank_approved_1639 | Ledipasvir |
| -11.4 | TheBindingDB_770 | Eptifibatide | -10.6 | DrugBank_approved_2002 | Elbasvir |
| -11.3 | dbfda-world_176 | Dihydroergotamine | | | |
| -11.3 | DrugBank_approved_1401 | Icatibant | | | |

| TMPRSS2_1-S1/S2_2 | | | TMPRSS2_2-S1/S2_2 | | |
|---|---|---|---|---|---|
| **Energy** | **Database** | **Name** | **Energy** | **Database** | **Name** |
| -12.3 | dbfda-world_531 | Amphotericin B | -12.6 | DrugBank_approved_980 | Candicidin |
| -12.3 | DrugBank_approved_980 | Candicidin | -12.3 | dbfda-world_531 | Amphotericin B |
| -12.2 | dbfda-world_496 | Nystatin | -12.1 | DrugBank_approved_1137 | Quinupristin |
| -11.7 | dbfda-world_494 | Nystatin | -11.8 | dbfda-world_256 | Digoxin |
| -11.6 | DrugBank_approved_506 | Nystatin | -11.7 | dbfda-world_493 | Nystatin |
| -11.5 | dbfda-world_493 | Nystatin | -11.1 | DrugBank_approved_506 | Nystatin |
| -11.5 | DrugBank_approved_1814 | Tannic acid | -11 | DrugBank_approved_2021 | Velpatasvir |
| -11.3 | dbfda-world_1534 | Paritaprevir | -11 | dbfda-world_494 | Nystatin |
| -11.3 | DrugBank_approved_2108 | Cantharidin | -10.9 | DrugBank_approved_2108 | Cantharidin |
| -11.3 | dbfda-world_1519 | Trypan blue | -10.8 | dbfda-world_495 | Nystatin |
| -11.2 | DrugBank_approved_2079 | Entrectinib | -10.8 | dbfda-world_496 | Nystatin |
| -11.2 | dbfda-world_495 | Nystatin | -10.7 | dbfda-world_892 | Rifampicin |
| -11.1 | DrugBank_approved_2005 | Venetoclax | -10.6 | dbfda-world_1136 | Everolimus |
| -11 | DrugBank_approved_3 | Desmopressin | -10.5 | dbfda-world_1028 | Rifapentine |
| -11 | dbfda-world_256 | Digoxin | -10.4 | dbfda-world_176 | Dihydroergotamine |
| -11 | dbfda-world_1028 | Rifapentine | -10.4 | DrugBank_approved_1639 | Ledipasvir |
| -10.9 | dbfda-world_1621 | Venetoclax | -10.3 | dbfda-world_690 | Natamycin |
| -10.9 | dbfda-world_690 | Natamycin | -10.3 | DrugBank_approved_3 | Desmopressin |
| -10.9 | TheBindingDB_660 | Ivacaftor | -10.3 | DrugBank_approved_2005 | Venetoclax |
| -10.8 | DrugBank_approved_1137 | Quinupristin | -10.3 | dbfda-world_545 | Ergotamine |
| -10.8 | TheBindingDB_770 | Eptifibatide | -10.2 | dbfda-world_1621 | Venetoclax |
| -10.8 | DrugBank_approved_2021 | Velpatasvir | -10.2 | dbfda-world_735 | Sirolimus |

| TMPRSS2_1-S2' | | | TMPRSS2_2-S2' | | |
|---|---|---|---|---|---|
| **Energy** | **Database** | **Name** | **Energy** | **Database** | **Name** |
| -12.1 | DrugBank_approved_2108 | Cantharidin | -13.7 | DrugBank_approved_1814 | Tannic acid |
| -10.9 | dbfda-world_256 | Digoxin | -13.2 | DrugBank_approved_8 | Abarelix |
| -10.7 | ChemBridge_26 | 7786009 | -12.8 | dbfda-world_256 | Digoxin |
| -10.6 | DrugBank_approved_1361 | Indacaterol | -12.7 | DrugBank_approved_2441 | Ubrogepant |
| -10.4 | TheBindingDB_910 | Avagard | -12.6 | dbfda-world_730 | Conivaptan |
| -10.4 | TheBindingDB_174 | Raltegravir | -12.5 | DrugBank_approved_2005 | Venetoclax |
| -10.3 | TheBindingDB_87 | Nexavar | -12.3 | dbfda-world_176 | Dihydroergotamine |
| -10.3 | dbfda-world_494 | Nystatin | -12.2 | DrugBank_approved_90 | Adapalene |
| -10.2 | dbfda-world_730 | Conivaptan | -12.2 | TheBindingDB_955 | Tasigna |
| -10.1 | DrugBank_approved_2058 | Bictegravir | -12.1 | TheBindingDB_1320 | Tannic Acid |
| -10 | DrugBank_approved_1076 | Gliquidone | -12.1 | TheBindingDB_1061 | Vapsirol |
| -10 | DrugBank_approved_2274 | Netarsudil | -12 | dbfda-world_75 | Adapalene |
| -10 | TheBindingDB_1139 | Regorafenib | -12 | DrugBank_approved_720 | Conivaptan |
| -10 | TheBindingDB_688 | Irbesartan | -11.9 | dbfda-world_1573 | Ecamsule |
| -9.9 | DrugBank_approved_2030 | Delamanid | -11.9 | DrugBank_approved_1162 | Antrafenine |
| -9.9 | DrugBank_approved_1694 | Daclatasvir | -11.9 | TheBindingDB_408 | Vapsirol |
| -9.9 | DrugBank_approved_377 | Acetyldigitoxin | -11.9 | TheBindingDB_402 | Synarel |

| | | | | | |
|---|---|---|---|---|---|
| -9.9 | ChemBridge_10 | 7793889 | -11.9 | TheBindingDB_772 | Tolvaptan |
| -9.8 | dbfda-world_1085 | Paliperidone | -11.8 | dbfda-world_1455 | Ledipasvir |
| -9.9 | - | Camostat | -11.8 | dbfda-world_1396 | Lomitapide |

| **catL_1-S1/S2′_1⁺** | | | **catL_2-S1/S2′_1⁺** | | | **catL_3-S1/S2′_1⁺** | | |
|---|---|---|---|---|---|---|---|---|
| **Energy** | **Database** | **Name** | **Energy** | **Database** | **Name** | **Energy** | **Database** | **Name** |
| -11.1 | DrugBank_approved_980 | Candicidin | -11.9 | dbfda-world_1572 | Ecamsule | -11.4 | dbfda-world_531 | Amphotericin B |
| -11 | dbfda-world_494 | Nystatin | -11.8 | dbfda-world_1573 | Ecamsule | -11.3 | dbfda-world_496 | Nystatin |
| -10.6 | dbfda-world_256 | Digoxin | -11.4 | dbfda-world_632 | Irinotecan | -11.2 | dbfda-world_256 | Digoxin |
| -10.4 | DrugBank_approved_1137 | Quinupristin | -11.4 | DrugBank_approved_2108 | Cantharidin | -10.7 | DrugBank_approved_1137 | Quinupristin |
| -10.2 | DrugBank_approved_506 | Nystatin | -11.4 | dbfda-world_256 | Digoxin | -10.7 | dbfda-world_495 | Nystatin |
| -10.2 | dbfda-world_495 | Nystatin | -11.3 | dbfda-world_176 | Dihydroergotamine | -10.6 | DrugBank_approved_506 | Nystatin |
| -10.1 | DrugBank_approved_3 | Desmopressin | -11.2 | dbfda-world_730 | Conivaptan | -10.4 | DrugBank_approved_540 | Amphotericin B |
| -10.1 | dbfda-world_496 | Nystatin | -11.2 | dbfda-world_1428 | Ponatinib | -10.3 | DrugBank_approved_980 | Candicidin |
| -10 | dbfda-world_531 | Amphotericin B | -11.2 | dbfda-world_1519 | Trypan blue | -10.3 | dbfda-world_494 | Nystatin |
| -10 | dbfda-world_545 | Ergotamine | -11.2 | TheBindingDB_511 | Accolate | -10.2 | DrugBank_approved_1401 | Icatibant |
| -9.8 | dbfda-world_176 | Dihydroergotamine | -11.1 | DrugBank_approved_2079 | Entrectinib | -10.1 | DrugBank_approved_3 | Desmopressin |
| -9.8 | dbfda-world_1634 | Deflazacort | -11.1 | dbfda-world_1263 | Eltrombopag | -10.1 | DrugBank_approved_1639 | Ledipasvir |
| -9.8 | DrugBank_approved_2005 | Venetoclax | -11 | dbfda-world_1265 | Tolvaptan | -10 | DrugBank_approved_1339 | Nilotinib |
| -9.8 | dbfda-world_1028 | Rifapentine | -10.9 | DrugBank_approved_555 | Ergotamine | -9.9 | DrugBank_approved_555 | Ergotamine |
| -9.7 | dbfda-world_1534 | Paritaprevir | -10.9 | dbfda-world_1425 | Regorafenib | -9.9 | TheBindingDB_955 | Tasigna |
| -9.7 | DrugBank_approved_811 | Dactinomycin | -10.8 | dbfda-world_1075 | Retapamulin | -9.9 | TheBindingDB_1061 | Vapsirol |
| -9.7 | DrugBank_approved_2108 | Cantharidin | -10.8 | DrugBank_approved_1670 | Edoxaban | -9.9 | DrugBank_approved_1046 | Rifaximin |
| -9.6 | TheBindingDB_435 | Fluorometholone | -10.8 | DrugBank_approved_720 | Conivaptan | -9.8 | TheBindingDB_408 | Vapsirol |
| -9.6 | DrugBank_approved_555 | Ergotamine | -10.7 | DrugBank_approved_125 | Ziprasidone | -9.8 | dbfda-world_493 | Nystatin |
| | | | -10.7 | DrugBank_approved_2020 | Lifitegrast | -9.7 | DrugBank_approved_2079 | Entrectinib |
| | | | -10.7 | DrugBank_approved_2005 | Venetoclax | -9.7 | DrugBank_approved_1814 | Tannic acid |

| **catL_1-S1/S2′_2⁺** | | | **catL_2-S1/S2′_2⁺** | | | **catL_3-S1/S2′_2⁺** | | |
|---|---|---|---|---|---|---|---|---|
| **Energy** | **Database** | **Name** | **Energy** | **Database** | **Name** | **Energy** | **Database** | **Name** |
| -11.1 | dbfda-world_531 | Amphotericin B | -11.4 | dbfda-world_1263 | Eltrombopag | -12 | dbfda-world_494 | Nystatin |
| -11.1 | dbfda-world_256 | Digoxin | -11.3 | dbfda-world_256 | Digoxin | -11.2 | dbfda-world_531 | Amphotericin B |
| -10.6 | dbfda-world_493 | Nystatin | -11.1 | dbfda-world_730 | Conivaptan | -11 | dbfda-world_495 | Nystatin |
| -10.3 | dbfda-world_1519 | Trypan blue | -10.9 | TheBindingDB_780 | Juxtapid | -10.9 | DrugBank_approved_1137 | Quinupristin |
| -10.3 | DrugBank_approved_980 | Candicidin | -10.7 | dbfda-world_1629 | Velpatasvir | -10.9 | DrugBank_approved_980 | Candicidin |
| -10.2 | DrugBank_approved_1137 | Quinupristin | -10.7 | TheBindingDB_955 | Tasigna | -10.7 | dbfda-world_1048 | Rifaximin |
| -10.2 | dbfda-world_176 | Dihydroergotamine | -10.7 | dbfda-world_1534 | Paritaprevir | -10.7 | DrugBank_approved_725 | Sirolimus |
| -10.1 | dbfda-world_1534 | Paritaprevir | -10.6 | DrugBank_approved_1409 | Eltrombopag | -10.5 | DrugBank_approved_2026 | Temoporfin |
| -10.1 | dbfda-world_495 | Nystatin | -10.6 | dbfda-world_496 | Nystatin | -10.5 | dbfda-world_496 | Nystatin |
| -10.1 | dbfda-world_494 | Nystatin | -10.5 | DrugBank_approved_2079 | Entrectinib | -10.5 | dbfda-world_256 | Digoxin |
| -10 | DrugBank_approved_1814 | Tannic acid | -10.5 | TheBindingDB_989 | Promacta | -10.4 | DrugBank_approved_555 | Ergotamine |
| -9.9 | DrugBank_approved_3 | Desmopressin | -10.5 | dbfda-world_494 | Nystatin | -10.4 | dbfda-world_1534 | Paritaprevir |
| -9.8 | DrugBank_approved_2265 | Pibrentasvir | -10.4 | dbfda-world_495 | Nystatin | -10.4 | dbfda-world_493 | Nystatin |
| -9.8 | dbfda-world_1621 | Venetoclax | -10.4 | DrugBank_approved_1639 | Ledipasvir | -10.3 | dbfda-world_1654 | Glecaprevir |
| -9.8 | DrugBank_approved_377 | Acetyldigitoxin | -10.4 | DrugBank_approved_2005 | Venetoclax | -10.3 | dbfda-world_1299 | Midostaurin |
| -9.8 | dbfda-world_496 | Nystatin | -10.3 | DrugBank_approved_1814 | Tannic acid | -10.2 | DrugBank_approved_3 | Desmopressin |
| -9.8 | DrugBank_approved_1543 | Triptorelin | -10.3 | DrugBank_approved_1401 | Icatibant | -10.2 | DrugBank_approved_1639 | Ledipasvir |
| -9.8 | DrugBank_approved_2108 | Cantharidin | -10.3 | dbfda-world_1529 | Lumacaftor | -10.2 | dbfda-world_1519 | Trypan blue |
| -9.8 | dbfda-world_1028 | Rifapentine | -10.3 | DrugBank_approved_479 | Imatinib | -10.1 | dbfda-world_176 | Dihydroergotamine |
| -9.7 | dbfda-world_1572 | Ecamsule | -10.3 | DrugBank_approved_2108 | Cantharidin | -10.1 | dbfda-world_736 | Sirolimus |
| | | | | | | -10 | dbfda-world_1027 | Bromocriptine |

*Unit of energy is kcal/mol. AutoDock Vina (version 1.1.2, Linux) was used for the docking.
+ S1/S2′ corresponds to the cleavage site targeted by catL (T696-M697) on S1/S2 boundary.

## **Table S3.** The average binding free energy of protein-ligand complexes*

| Complex | $\Delta G$ | Complex | $\Delta G$ | Complex | $\Delta G$ |
|---|---|---|---|---|---|
| trypsin-S1/S2-Digoxin | -189.21922 | TMPRSS2-S2'-Ubrogepant | -44.6749 | catL-Digoxin | 468.1419 |
| trypsin-S1/S2-FAD | -186.88962 | S1/S2-Ciclesonide | -40.2 | catL-S1/S2'-Irinotecan | 469.175 |
| S1/S2-Avatrombopag | -154.4893 | trypsin-Digoxin | -22.3252 | catL-S1/S2'-Vapsirol | 477.135 |
| trypsin-S2'-Ursodeaoxycholic acid | -154.29806 | catL-Nystatin | -17.5213 | S2'-D936Y-Saquinavir | 490.7611 |
| TMPRSS2-S1/S2-Digoxin | -147.1056 | TMPRSS2-Nystatin | -8.97008 | trypsin-Dutasteride | 545.7077 |
| catL-S1/S2'-Rifapentine | -138.49152 | S1/S2-Nystatin | -4.07952 | S1/S2-Rifaximin | 574.1273 |
| trypsin-S1/S2-Adapalene | -121.2825 | trypsin-S2'-Dihydroergotamine | 0.9803 | trypsin-S1/S2-Bictegravir | 579.2934 |
| TMPRSS2-S1/S2-Telmisartan | -121.09444 | S1/S2-Capmatinib | 29.65224 | trypsin-Digoxin | 594.7297 |
| catL-Digoxin | -120.32792 | S1/S2'-Ubrogepant | 31.30914 | S1/S2-Ubrogepant | 608.25448 |
| S2'-Antrafenine | -118.58336 | catL-S1/S2'-Nystatin | 40.80676 | trypsin-S2'-Digoxin | 608.7868 |
| TMPRSS2-S1/S2-Digoxin | -116.02408 | catL-Digoxin | 45.23074 | trypsin-S1/S2-Dihydroergotamine | 613.8832 |
| catL-S1/S2'-Rifapentine | -114.56038 | trypsin-Valrubicin | 52.70146 | S1/S2'-Nystatin | 621.6618 |
| trypsin-S2'-Lasmiditan | -109.25438 | TMPRSS2-Sirolimus | 67.8708 | t1-S2'-D936Y-Nebivolol | 640.6687 |
| trypsin-S2'-Glimepiride | -107.87552 | TMPRSS2-Digoxin | 74.04584 | TMPRSS2-S2'-Dihydroergotamine | 641.7708 |
| TMPRSS2-S2'-Bictegravir | -104.40558 | TMPRSS2-Drospirenone | 83.41938 | trypsin-S1/S2-Dihydroergotamine | 644.3816 |
| catL-Drospirenone | -101.37212 | catL-Rifaximin | 91.25842 | TMPRSS2-S2'-Digoxin | 647.4213 |
| TMPRSS2-Dexamethasone M | -99.6151 | S2'-D936Y-Glyxambi | 95.0251 | TMPRSS2-Ubrogepant | 657.21852 |
| trypsin-S1/S2-Ritonavir | -99.27076 | trypsin-Digoxin | 109.0991 | trypsin-S2'-Nystatin | 710.1351 |
| S2'-D936Y-Irbesartan | -95.90374 | trypsin-S1/S2-Droperidol | 110.7961 | catL-S1/S2'-Edoxaban | 714.1895 |
| TMPRSS2-Digoxin | -95.62794 | S1/S2-Vapsirol | 111.637 | catL-S1/S2'-Digoxin | 719.4035 |
| S2'-D936Y-Nebivolol | -94.06474 | catL-S1/S2'-Pibrentasvir | 111.949 | trypsin-S2'-Aprepitant | 725.1868 |
| trypsin-S1/S2-Drospirenone | -93.28244 | S2'-D936Y-Deoxycholic acid | 113.3931 | catL-S1/S2'-Drospirenone | 736.8676 |
| catL-S1/S2'-Deflazacort | -91.99702 | S2'-D936Y-Viracept | 133.7918 | catL-Dihydroergotamine | 817.161 |
| TMPRSS2-S2'-Gliquidone | -90.84554 | trypsin-S1/S2-Dihydroergotamine | 143.2903 | trypsin-S1/S2-Dihydroergotamine | 845.0419 |
| TMPRSS2-S2'-Antrafenine | -90.32412 | trypsin-S1/S2-Antrafenine | 151.7891 | trypsin-S1/S2-Gleevec | 848.3642 |
| TMPRSS2-S1/S2-Nystatin | -87.9875 | TMPRSS2-S1/S2-Elbasvir | 152.1473 | S1/S2-Digoxin | 870.507 |
| S1/S2'-Digoxin | -86.89394 | trypsin-S1/S2-Capmatinib | 170.6533 | S2'-Digoxin | 891.34192 |
| catL-S1/S2'-Rifaximin | -84.2909 | catL-S1/S2'-Dihydroergotamine | 170.7121 | trypsin-S2'-Antrafenine | 915.6934 |
| TMPRSS2-Ubrogepant | -83.68272 | TMPRSS2-S1/S2-Dihydroergotamine | 176.0496 | t2-S2'-D936Y-Atovaquone | 991.0772 |
| catL-S1/S2'-Digoxin | -83.00332 | S1/S2-Dihydroergotamine | 178.0787 | trypsin-Dihydroergotamine | 994.1542 |
| trypsin-S1/S2-Digoxin | -81.86608 | catL-S1/S2'-Fluorop | 187.3583 | TMPRSS2-S2'-Daclatasvir | 1050.959 |
| S1/S2-Cefpiramide | -81.200879 | catL-S1/S2'-Dihydroergotamine | 200.0495 | catL-Dihydroergotamine | 1071.849 |
| catL-Rifapentine | -81.06458 | TMPRSS2-S2'-Adapalene | 215.1768 | trypsin-Saquinavir | 1113.378 |
| trypsin-Saquinavir | -80.90818 | t2-S2'-D936Y-Paliperidone | 215.225959 | TMPRSS2-S1/S2-Drospirenone | 1120.802 |
| S1/S2'-Rifapentine | -80.62622 | t1-S2'-D936Y-Rolapitant | 228.956 | TMPRSS2-S1/S2-Sirolimus | 1123.432 |
| trypsin-S1/S2-Capmatinib | -75.69074 | trypsin-S2'-Raltegravir | 229.0492 | catL-S1/S2'-Dihydroergotamine | 1150.167 |
| TMPRSS2-S1/S2-Nystatin | -74.57538 | trypsin-S2'-Axinitib | 229.4983 | S1/S2-Digoxin | 1252.084 |
| S1/S2-Digoxin | -74.53658 | trypsin-S2'-Vapsirol | 230.2532 | S2'-Drospirenone | 1339.57034 |
| TMPRSS2-Saquinavir | -73.14612 | trypsin-S1/S2-Nystatin | 233.5296 | S1/S2'-Drospirenone | 1371.384 |
| S1/S2'-Rifaximin | -71.57796 | TMPRSS2-Dihydroergotamine | 237.2782 | trypsin-S1/S2-Vemurafenib | 1392.289 |
| trypsin-Drospirenone | -70.2387 | trypsin-S2'-Vapsirol | 247.7259 | TMPRSS2-S2'-Digoxin | 1411.963 |
| S1/S2'-Dihydroergotamine | -69.52424 | TMPRSS2-S1/S2-Simeprevir | 253.0695 | catL-S1/S2'-Digoxin | 1543.352 |
| S1/S2'-Capmatinib | -68.23316 | S1/S2-Vapsirol | 269.363 | catL-S1/S2'-Nystatin | 1575.642 |
| t2-S2'-D936Y-Risperidone | -67.53934 | TMPRSS2-Vapsirol | 272.8234 | trypsin-S2'-Elbasvir | 1634.338 |
| S1/S2'-Digoxin | -67.3627 | trypsin-S2'-Digoxin | 286.9939 | TMPRSS2-S2'-Delamanid | 1704.878 |
| trypsin-Rifapentine | -67.03428 | S2'-D936Y-Atovaquone | 298.3715 | trypsin-S2'-Viibryd | 1725.864 |
| trypsin-S1/S2-Ubrogepant | -64.71386 | catL-Dihydroergotamine | 311.9322 | TMPRSS2-S2'-Irbesartan | 1757.756 |
| trypsin-S1/S2-Digoxin | -63.72308 | catL-S1/S2'-Promacta | 313.5024 | trypsin-S1/S2-Rifapentine | 1810.328 |
| TMPRSS2-S2'-Vapsirol | -63.36442 | trypsin-S1/S2-Digoxin | 385.2007 | TMPRSS2-S1/S2-Dihydroergotamine | 2025.632 |
| TMPRSS2-S1/S2-Nystatin | -63.1792 | TMPRSS2-Dexamethasone M | 388.51754 | catL-S1/S2'-Digoxin | 2417.164 |
| t1-S2'-D936Y-Olaparib | -62.88132 | TMPRSS2-S1/S2-Rifapentine | 390.1107 | S1/S2'-Digoxin | 2422.388 |
| trypsin-S2'-Prelay | -61.45806 | trypsin-S1/S2-Rifapentine | 405.8055 | trypsin-S1/S2-Digoxin | 2458.327 |
| catL-S1/S2'-Nystatin | -61.40966 | trypsin-S1/S2-Ilevro | 408.7774 | TMPRSS2-S2'-Raltegravir | 2485.941 |
| TMPRSS2-S1/S2-Dihydroergotamine | -61.17566 | trypsin-S1/S2-Diosmin | 420.0925 | TMPRSS2-S2'-Indacaterol | 2553.111 |
| trypsin-S1/S2-Nystatin | -54.27652 | trypsin-S1/S2-Ibrutinib | 423.8819 | TMPRSS2-S1/S2-Rifapentine | 2589.211 |
| trypsin-S1/S2-Dihydroergotamine | -51.3636 | TMPRSS2-S1/S2-Digoxin | 426.7328 | trypsin-Nystatin | 3320.98 |
| catL-S1/S2'-Dihydroergotamine | -49.38546 | TMPRSS2-S1/S2-Digoxin | 463.0654 | TMPRSS2-Digoxin | 4537.587 |
| S1/S2-Dihydroergotamine | -46.31082 | catL-S1/S2'-Tolvaptan | 463.8297 | | |

*Unit of $\Delta G$ is kJ/mol.

**Table S4.** Average number of contacts between residues of catL in catL-ligand interactions, defined by a distance cut-off of 6Å in the 100ns simulation trajectories. Residues with at least one contact (on an average) to the ligand are listed. Catalytic residues are highlighted with red and residues in close vicinity to catalytic sites are highlighted with blue.

| | Average number of contacts | | | |
|---|---|---|---|---|
| | **Rifapentine** | **Drospirenone** | **Digoxin** | **Nystatin** |
| K17 | | | 1.01 | |
| N18 | 7.65 | 3.36 | 7.06 | 2.61 |
| Q19 | 4.64 | 6.91 | 8.13 | 5.33 |
| G20 | 3.60 | 3.86 | 3.73 | 3.64 |
| Q21 | 4.45 | 8.43 | 5.15 | 8.87 |
| **C22** | | 4.10 | | 5.63 |
| **G23** | | 2.53 | | 3.80 |
| W26 | | | 1.55 | |
| **C65** | | | | 5.58 |
| **N66** | | | | 6.81 |
| E87 | 1.69 | | | |
| P90 | 1.29 | | | |
| Y91 | 7.51 | | 1.95 | 3.41 |
| Q92 | 3.62 | | | 3.36 |
| A93 | 2.99 | | | 3.34 |
| T94 | | | | 4.02 |
| E141 | 1.86 | | | |
| L144 | 3.77 | 5.32 | 7.28 | 4.51 |
| F145 | 3.09 | | 4.23 | 2.01 |
| **D162** | | | 1.11 | |
| **H163** | | 1.39 | 1.80 | |
| S188 | | 1.33 | 1.68 | |
| W189 | 7.16 | 13.67 | 13.79 | 12.51 |
| G190 | 1.31 | 2.54 | 2.32 | |
| E192 | 2.29 | 6.12 | 2.64 | |
| W193 | 1.25 | 7.33 | 5.98 | 2.63 |

**Table S5.** The occupancy percentage of catL-ligand interactions. Catalytic residues are highlighted with red and residues in close vicinity to catalytic sites are highlighted with blue. The calculations were performed for the whole 100ns simulation trajectories.

| | **The occupancy percentage of the interactions** | | | |
|---|---|---|---|---|
| | **Rifapentine** | **Drospirenone** | **Digoxin** | **Nystatin** |
| N18 | 99.6% | 94.9% | 95.5% | 82.2% |
| Q19 | 99.5% | 99.2% | 99.9% | 99.6% |
| G20 | 99.5% | 99.2% | 96.6% | 99.1% |
| Q21 | 92.9% | 99.4% | 91.8% | 99.5% |
| **C22** | | 98.1% | | 99.7% |
| **G23** | | 94.7% | | 99.9% |
| **S24** | | | | 71.0% |
| **C65** | | | | 99.2% |
| **N66** | | | | 97.8% |
| Y91 | 99.8% | | | |
| Q92 | 90.8% | | | 70.6% |
| A93 | 99.1% | | | 80.6% |
| T94 | | 99.0% | | 83.3% |
| L144 | | | 100% | 95.5% |
| F145 | | | 78.7% | |
| S188 | | 81.5% | 88.5% | |
| W189 | | 99.1% | 100% | 98.0% |
| G190 | | 86.9% | 89.0% | |
| E192 | | 92.5% | 74.4% | |
| W193 | | 97.3% | 95.3% | |

**Table S6.** The grid parameters for all structures used in the docking

| Structure | center_x | center_y | center_z | size_x | size_y | size_z |
|---|---|---|---|---|---|---|
| trypsin_1 | -7.129 | -1.032 | -8.192 | 32 | 32 | 26 |
| trypsin_3 | -7.77 | -1.28 | -9.3 | 24 | 40 | 26 |
| trypsin_2 | -8.77 | -1.28 | -9.3 | 24 | 40 | 26 |
| catL_3 | -3.08 | -9.84 | -6.49 | 48 | 48 | 54 |
| catL_2 | -5.2 | -9.84 | -6.49 | 48 | 42 | 54 |
| catL_1 | -5.2 | -9.84 | -6.49 | 48 | 48 | 54 |
| tmprss2_3 | 11.106 | 1.809 | 0.84 | 22 | 32 | 54 |
| tmprss2_2 | 11.106 | 1.809 | 0.84 | 22 | 32 | 54 |
| tmprss2_1 | 11.106 | 1.809 | 0.84 | 22 | 32 | 54 |
| t3-s2p_d936y | 77.77 | 27.55 | 35.81 | 82 | 40 | 56 |
| t2-s2p_d936y | 88.47 | 31.22 | 32.06 | 78 | 36 | 60 |
| t1-s2p_d936y | 77.42 | 26.13 | 24.42 | 62 | 40 | 52 |
| c3-s1 | 1.81 | -1.04 | 0.48 | 24 | 52 | 44 |
| c1-s1 | 1.81 | -1.04 | 0.48 | 24 | 52 | 44 |
| tm2-s2 | 7.02 | -1.04 | -2.66 | 34 | 56 | 60 |
| tm1-s1 | 4.41 | 1.85 | 1.92 | 36 | 56 | 60 |
| t3-s1 | 0 | 0.69 | 5.91 | 26 | 50 | 40 |
| t2-s1 | 0 | 0.69 | 5.91 | 26 | 50 | 40 |
| t1-s1 | 0 | 0.69 | 5.91 | 26 | 50 | 40 |
| s12_3 | 20.91 | -10.54 | 7.86 | 40 | 40 | 24 |
| s12_1 | 7.76 | -15.99 | 3.67 | 40 | 40 | 24 |
| c2-s2 | 1.81 | -1.04 | 0.48 | 24 | 52 | 44 |
| tm2-s1 | 1.1 | 1.85 | 1.92 | 36 | 56 | 60 |
| s2p_d936y | 71.49 | 29.68 | 26.72 | 40 | 40 | 40 |
| s12_2p | 11.34 | 19.3 | 10.79 | 40 | 28 | 34 |
| c2-s1 | 1.81 | -1.04 | 0.48 | 24 | 52 | 44 |
| tm1-s2p | -10.32 | -1.04 | -2.66 | 32 | 56 | 60 |
| t3-s2p | -9.84 | 4.91 | 1.92 | 60 | 24 | 60 |
| t2-s2p | -10.61 | -3.56 | 1.92 | 30 | 62 | 60 |
| t1-s2p | -14.14 | -3.76 | 1.92 | 62 | 40 | 52 |
| s2p | 65.54 | 28.87 | 33.2 | 54 | 40 | 28 |
| s12_2 | 11.34 | -15.99 | 10.79 | 40 | 40 | 24 |
| c3-s2 | 1.81 | -1.04 | 0.48 | 24 | 52 | 44 |
| c1-s2 | 1.81 | -1.04 | 0.48 | 24 | 52 | 44 |
| tm2-s2p | -3.48 | -1.04 | -2.66 | 62 | 28 | 60 |
| tm1-s2 | 7.02 | -1.04 | -2.66 | 34 | 56 | 60 |
| t3-s2 | 0 | -2.29 | 1.92 | 26 | 50 | 52 |
| t2-s2 | 0 | -2.29 | 1.92 | 26 | 50 | 52 |
| t1-s2 | 0 | 0.69 | 1.92 | 26 | 50 | 52 |
| s12_3p | 11.34 | 19.3 | 5.39 | 40 | 28 | 34 |
| s12_1p | 7.76 | 19.3 | 5.39 | 40 | 28 | 34 |

**Table S7.** Acute toxicity of drugs

| Drug | LD50 (mg/kg/day) | Drug | LD50 (mg/kg/day) | Drug | LD50 (mg/kg/day) |
|---|---|---|---|---|---|
| Abarelix | 5 | Icatibant | 760 | Ubrogepant | 50 |
| Accolate | 1600 | Imatinib | 120 | Ursodeoxycholic acid | 10000 |
| Acetyldigitoxin | 7.3 | Indacaterol | 2000 | Valrubicin | 109 |
| Adapalene | 5000 | Irbesartan | 2000 | Vapsirol | 500 |
| Alimta | 15 | Irinotecan | 765 | Velpatasvir | 300 |
| Amphotericin B | 5000 | Ivacaftor | 400 | Vemurafenib | 250 |
| Antrafenine | 4000 | Ivermectin | 25 | Venetoclax | 5 |
| Aprepitant | 1000 | Juxtapid | 10 | Viibryd | 200 |
| Atovaquone | 20 | Lapatinib | 10 | Vontrol | 450 |
| Avatrombopag | 160 | Lasmiditan | 200 | Ziprasidone | 200 |
| Axitinib | 2000 | Ledipasvir | 300 | | |
| B.H And Epinephrine | 50 | Lifitegrast | 10 | | |
| Bacitracin | 3750 | Lomitapide | 1.5 | | |
| Bictegravir | 300 | Lumacaftor | 1000 | | |
| Bromocriptine | 800 | Mellaril-S | 385 | | |
| Cafergot | 300 | Midostaurin | 200 | | |
| Camptosar | 765 | Nafarelin | 50 | | |
| Canagliflozin | 30 | Naldemedine | 720 | | |
| Candesartan cilexetil | 2000 | Natamycin | 1500 | | |
| Candicidin | 1000 | Nepafenac | 2000 | | |
| Cantharidin | 75 | Netarsudil | 45 | | |
| Capmatinib | 2000 | Nexavar | 10 | | |
| Cefpiramide | 12500 | Nilotinib | 8 | | |
| Cetrorelix | 68 | Nupercaine | 50 | | |
| Ciclesonide | 0.5 | Nystatin | 8000 | | |
| Concentraid | 0.0015 | Olaparib | 15 | | |
| Conivaptan | 0.5 | Oxytocin | 514 | | |
| D.H.E. 45 | 2000 | Paclitaxel | 128 | | |
| Daclatasvir | 370 | Paliperidone | 65 | | |
| Dactinomycin | 13 | Paritaprevir | 30 | | |
| Daptomycin | 6 | Pazopanib | 5.4 | | |
| Deflazacort | 5200 | Pexidartinib | 10 | | |
| Delamanid | 150 | Pibrentasvir | 600 | | |
| Desmopressin | 0.2 | Plecanatide | 600 | | |
| Dexamethasone M | 873 | Ponatinib | 3 | | |
| Digoxin | 17.8 | Posaconazole | 30 | | |
| Dihydroergotamine | 8000 | Prelay | 5000 | | |
| Diosmin | 3000 | Promacta | 150 | | |
| Docetaxel | 156 | Quinupristin | 7.5 | | |
| Droperidol | 160 | Raltegravir | 600 | | |
| Drospirenone | 10 | Rapamune | 2500 | | |
| Dutasteride | 2000 | Regorafenib | 1 | | |
| Duvelisib | 25 | Retapamulin | 2300 | | |
| Ecamsule | 1000 | Revefenacin | 0.5 | | |
| Edoxaban | 1000 | Rifampicin | 500 | | |
| Elbasvir | 1000 | Rifapentine | 3300 | | |
| Eltrombopag | 75 | Rifaximin | 2 | | |
| Entrectinib | 7.5 | Ritonavir | 2500 | | |
| Eplerenone | 20 | Rutin | 200 | | |
| Eptifibatide | 45 | Saquinavir | 10 | | |
| Ergotamine | 0.171 | Simeprevir | 1000 | | |
| Eribulin | 0.0283 | Sirolimus | 2500 | | |
| Estrone sulfate | 7000 | Solifenacin | 30 | | |
| Everolimus | 2000 | Synarel | 100 | | |

| | | | |
|---|---|---|---|
| Flavin adenine dinucleotide | 7000 | Tannic acid | 2260 |
| Fluorometholone | 2000 | Tasigna | 4 |
| Glecaprevir | 120 | Telmisartan | 3000 |
| Gleevec | 120 | Temoporfin | 0.3 |
| Glimepiride | 10000 | Tolvaptan | 2000 |
| Gliquidone | 15000 | Triamcinolone | 5000 |
| Ibrutinib | 1000 | Triptorelin | 400 |

## References


1. Studer, G.; Rempfer, C.; Waterhouse, A.M.; Gumienny, R.; Haas, J.; Schwede, T. QMEANDisCo-distance constraints applied on model quality estimation. *Bioinformatics* **2020** *36*, 2647.
2. Anderson, R.J.; Weng, Z.; Campbell, R.K.; Jiang, X. Main-chain conformational tendencies of amino acids. *Proteins* **2005**, *60*, 679–689.
